# Diagnosis and management of individuals with Fetal Valproate Spectrum Disorder; a consensus statement from the European Reference Network for Congenital Malformations and Intellectual Disability

**DOI:** 10.1186/s13023-019-1064-y

**Published:** 2019-07-19

**Authors:** Jill Clayton-Smith, Rebecca Bromley, John Dean, Hubert Journel, Sylvie Odent, Amanda Wood, Janet Williams, Verna Cuthbert, Latha Hackett, Neelo Aslam, Heli Malm, Gregory James, Lena Westbom, Ruth Day, Edmund Ladusans, Adam Jackson, Iain Bruce, Robert Walker, Sangeet Sidhu, Catrina Dyer, Jane Ashworth, Daniel Hindley, Gemma Arca Diaz, Myfanwy Rawson, Peter Turnpenny

**Affiliations:** 1 Division of Evolution and Genomic Sciences School of Biological Sciences, University of Manchester, Manchester, UK; 2 Manchester Centre for Genomic Medicine, Manchester University Hospitals NHS Foundation Trust, Manchester Academic Health Sciences Centre, Manchester, UK; 3 Paediatric Psychosocial Department, Royal Manchester Children’s Hospital, Manchester Academic Health Sciences Centre, Manchester, UK; 4 Clinical Genetics, Clinical Genetics Service, Ashgrove House, Foresterhill, Aberdeen, UK; 5 Génétique Médicale – Consultation, CHBA Centre Hospitalier Bretagne Atlantique - CH Chubert, 20 boulevard du Général Maurice Guillaudot, BP 70555, 56017 Vannes Cedex, France; 6 Service de Génétique Clinique, CNRS UMR 6290, Université de Rennes, CHU de Rennes - Hôpital Sud, 16 Boulevard de Bulgarie, 35203 Rennes Cedex 2, France; 7 Aston Brain Centre, School of Life and Health Sciences, Aston Triangle, Birmingham, UK; 8 Brain and Mind, Clinical Sciences, Murdoch Children’s Research Institute, Parkville, Melbourne, Australia; 9 INFACT/FACSA, Independent Fetal Anti-Convulsant Trust & FACS Syndrome Association, Preston, UK; 10 Department of Paediatric Rheumatology, Royal Manchester Children’s Hospital, Oxford Road, Manchester, M13 9WL UK; 11 Child and Adolescent Mental Health Services (CAMHS), Royal Manchester Children’s Hospital, Oxford Road, Manchester, M13 9WL UK; 12 Teratology Information Service, University of Helsinki and Department of Emergency Medicine and Services, Helsinki University Hospital, Tukholmankatu 17, 00029 HUS, Helsinki, Finland; 13 Department of Neurosurgery, Great Ormond Street Hospital, Great Ormond Street, London, UK; 14 Craniofacial Unit, Great Ormond Street Hospital, Great Ormond Street, London, WC1N 3JH UK; 15 Developmental Neurosciences, UCL Great Ormond Street Institute of Child Health, 30 Guilford Street, London, WC1N 1EH UK; 16 Victor Horsley Department of Neurosurgery, National Hospital for Neurology and Neurosurgery, Queen Square, London, WC1N 3BG UK; 17 Lund University, Barnmed klin, SUS, Lund, Sweden; 18 Guardian Medical Centre, Guardian Street, Warrington, UK; 19 Department of Paediatric Cardiology, Royal Manchester Children’s Hospital, Oxford Road, Manchester, UK; 20 Department of Neurology, Salford Royal Hospital NHS Trust, Stott Lane, Salford, UK; 21 Paediatric ENT Department, Royal Manchester Children’s Hospital, Manchester University NHS Foundation Trust, Manchester Academic Health Science Centre, Manchester, UK; 22 Department of Paediatric Anaesthesia, Royal Manchester Children’s Hospital, Oxford Road, Manchester, M13 9WL UK; 23 Department of Paediatric Nephrology, Royal Manchester Children’s Hospital, Oxford Road, Manchester, UK; 24 Cleft Lip and Palate Team, Royal Manchester Children’s Hospital, Oxford Road, Manchester, UK; 25 Manchester Royal Eye Hospital, Manchester University Hospitals NHS Foundation Trust, Manchester Academic Health Sciences Centre, Manchester, UK; 26 Community Paediatrics, Bolton NHS Foundation Trust, Breightmet Health Centre, Bolton, UK; 27 Department of Neonatology, Hospital Clinic (Maternitat), Sabino Arana 1, 08028 Barcelona, Spain; 28 Clinical Genetics, Royal Devon and Exeter NHS Foundation Trust, Gladstone Rd, Exeter, UK

**Keywords:** Fetal valproate syndrome, Expert consensus, Management, Guideline, Teratogen, Antiepileptic drug

## Abstract

**Background:**

A pattern of major and minor congenital anomalies, facial dysmorphic features, and neurodevelopmental difficulties, including cognitive and social impairments has been reported in some children exposed to sodium valproate (VPA) during pregnancy. Recognition of the increased risks of in utero exposure to VPA for congenital malformations, and for the neurodevelopmental effects in particular, has taken many years but these are now acknowledged following the publication of the outcomes of several prospective studies and registries. As with other teratogens, exposure to VPA can have variable effects, ranging from a characteristic pattern of major malformations and significant intellectual disability to the other end of the continuum, characterised by facial dysmorphism which is often difficult to discern and a more moderate effect on neurodevelopment and general health. It has become clear that some individuals with FVSD have complex needs requiring multidisciplinary care but information regarding management is currently lacking in the medical literature.

**Methods:**

An expert group was convened by ERN-ITHACA, the European Reference Network for Congenital Malformations and Intellectual Disability comprised of professionals involved in the care of individuals with FVSD and with patient representation. Review of published and unpublished literature concerning management of FVSD was undertaken and the level of evidence from these sources graded. Management recommendations were made based on strength of evidence and consensus expert opinion, in the setting of an expert consensus meeting. These were then refined using an iterative process and wider consultation.

**Results:**

Whilst there was strong evidence regarding the increase in risk for major congenital malformations and neurodevelopmental difficulties there was a lack of high level evidence in other areas and in particular in terms of optimal clinical management.. The expert consensus approach facilitated the formulation of management recommendations, based on literature evidence and best practice. The outcome of the review and group discussions leads us to propose the term Fetal Valproate Spectrum Disorder (FVSD) as we feel this better encompasses the broad range of effects seen following VPA exposure in utero.

**Conclusion:**

The expert consensus approach can be used to define the best available clinical guidance for the diagnosis and management of rare disorders such as FVSD. FVSD can have medical, developmental and neuropsychological impacts with life-long consequences and affected individuals benefit from the input of a number of different health professionals.

**Electronic supplementary material:**

The online version of this article (10.1186/s13023-019-1064-y) contains supplementary material, which is available to authorized users.

## Introduction and objectives to the consensus statement

Sodium valproate (VPA) is an effective antiepileptic drug (AED) which is also used to treat bipolar disorder, migraine and other mental health problems. It was first licensed for use in Europe in the mid 1970’s. In February 2018 the Pharmacovigilance Committee of the European Medicines Agency recommended that VPA should not be used in pregnancy unless the women concerned had seizures which were unresponsive to other drugs [1]. In April 2018, the Pharmacovigilance Committee of the European Medicines Agency recommended that women using VPA should use effective contraception and agreed that VPA use during pregnancy should be restricted to women with epilepsy who are unresponsive to other drugs [2]. In the forty years from when VPA use began in Europe, many women took this drug during pregnancy as it is an effective AED, often providing better seizure control than other AEDs [3]. From the early 1980’s reports began to be published suggesting that VPA exposure during pregnancy was associated with an increased risk for congenital malformations and poorer developmental trajectories [4–12]. As most of these were initially anecdotal, it was not until results became available from long term prospective cohort studies of children born to mothers taking VPA and from pregnancy registers [13–18], that the serious teratogenic effects of VPA were appropriately acknowledged. Currently available evidence suggests that the risk of congenital malformation after VPA exposure is around 11% [19, 20] but that the level of risk is associated with dose, with the risk being as high as 24% when the dose is over 1500 mg daily [20]. The risks to neurodevelopment appear diverse and depend on the specific domain (e.g. of cognition or behaviour) under study. There is replicated evidence of a reduction in IQ of 8–10 points compared to unexposed individuals and specific deficits in verbal skills [21, 22] as well as language impairment and poorer levels of daily living skills [23]. The prevalence of autism spectrum disorder (ASD) is 6–15% in VPA exposed individuals [24–26] which is greatly increased compared to the background population risk. In addition, a number of medical symptoms have been reported at increased frequency in valproate exposed individuals [27]. All of these features may combine to give a complex picture which requires multidisciplinary management requiring a large number of different professionals. Though we expect the number of individuals with valproate related difficulties to decline in future years due to changes in prescribing [28], there are still many children and adults living within Europe with the consequences of prenatal exposure to VPA. The number of affected children across the spectrum within the UK, for example, is estimated to be in excess of 20,000, based on the number of women prescribed VPA in pregnancy and the percentage of those estimated to show symptoms as a result, but no firm number is currently known. However, despite widespread use of VPA to treat epilepsy and bipolar disorder for a few decades the number of exposed children when viewed at a population level is likely to be relatively small, meaning that clinicians will have limited experience with the condition. Correct diagnosis and specialist management of this cohort is therefore important.

The objectives of this consensus statement are to provide guidance on the most effective management of the medical and neurodevelopmental problems of individuals demonstrating the effects of prenatal exposure to VPA from infancy to adulthood. This is undertaken in order to minimise health complications, to optimise the chances for individuals to achieve developmentally according to their potential, to enhance social adaptation, and to clarify the clinical diagnostic criteria for a disorder for which there remains no specific laboratory diagnostic test or biomarker. Whilst previous literature has referred to the effects of VPA exposure in utero as Fetal Valproate Syndrome [6, 10] we propose and use the term Fetal Valproate Spectrum Disorder (FVSD) to refer to the range of clinical and developmental effects attributed to exposure to VPA in utero. Further explanation of the use of this new term is given in the section on Diagnosis of Fetal Valproate Spectrum Disorder below.

This consensus statement was drawn up as an initiative of the European Reference Network, (ERN) ERN-ITHACA. The network encompasses several EU countries, with differing provision for healthcare, therapies and educational needs for individuals with developmental disorders. ERNs were established in 2017 as part of the EU Cross Border Health Care Directive after the responses to a survey of the needs of rare disease patients across Europe were taken into account [29]. We aimed for EU-wide coverage of this topic area when drawing up our recommendations so in some areas we have needed to be less specific and more general in our recommendations as a result. Our aim is that country-specific notes should be added to any future translations of this document.

## Target groups and questions to be covered

This document is intended for all health professionals including paediatricians, specialist paediatricians (e.g. nephrology, cardiology etc.) adult physicians and primary care physicians looking after the medical aspects of individuals with FVSD, for professionals involved in the diagnosis of FVSD and for those involved in management of the neurodevelopmental aspects of FVSD, including community paediatricians, educational psychologists, clinical psychologists, child psychiatrists and educators. It will also provide relevant information for professionals involved with managing the social aspects of the condition, (e.g. parents, social workers, support workers and occupational health physicians). The questions addressed by the group are listed in Table 1.

**Table 1:** Questions Addressed By the Consensus Group

1	What are the diagnostic criteria for Fetal Valproate Spectrum Disorder (FVSD)?
2	What investigations should be carried out in individuals with FVSD?
3	Are there any specific therapies which benefit individuals with FVSD?
4	What health and developmental surveillance should be undertaken in individuals with FVSD and by whom?
5	What neuropsychological assessments are needed for individuals with FVSD?
6	What school adjustments may be beneficial for children with FVSD?
7	What transition arrangements should be made for individuals with FVSD?
8	What medical checks should adults with FVSD have and who should undertake these?
9	What is the best format for the recommendations from the expert consensus group?

## Methodology for producing the expert consensus document

**Fig. 1:**
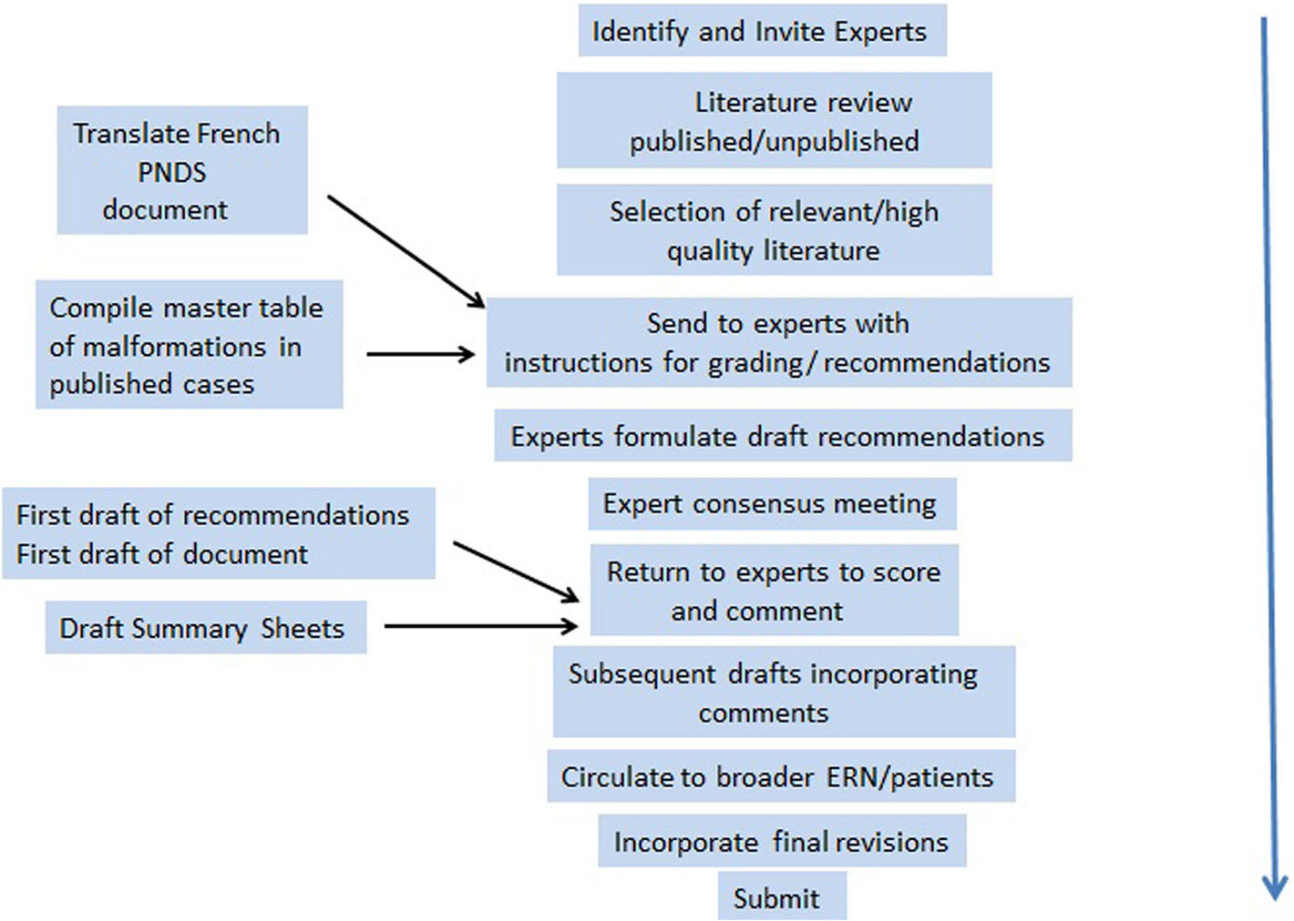
Outline Of The Process Of Producing The Consensus Statement

The overall process is outlined in Fig. 1. Peer-reviewed publications were ascertained by searching the PubMed and Cochrane databases. The search terms Fetal/Foetal and/or Valproate and/or Syndrome were combined with the terms management, diagnosis, growth, treatment, neural tube, skeletal, joint, limb, cardiac, gastrointestinal, feeding, genitourinary, renal, eye, ear, development, intellectual disability, medical, neurodevelopment, cognitive impairment, neuropsychology, autism spectrum, behaviour, neonatal, adult. We searched all articles from 1970 to 2017 but confined ourselves to papers published in English. A check for further relevant peer-reviewed articles was made by reviewing those ascertained by the French PNDS (National Protocol for Diagnosis and Care) document published in 2017 [30]. This document was compiled following the search recommendations of the Haute Autorité de Santé [31]. We did not identify any additional articles through this process but we translated the PNDS document into English and, after assessing the recommendations contained therein using the AGREE II tool [32] against which they compared favourably, the translated document was used as an additional resource for our working group. We also assessed information from meeting abstracts, from our own unpublished data, and from reviews and book chapters on the topic known to members of the expert group because of their interest in the field over a long period of time. We compiled a table of all of the individual case reports of individuals with congenital malformations after VPA exposure in the literature. We considered the strengths and limitations of the body of evidence obtained; there have been no randomised controlled trials (RCTs) carried out in this area because once adverse effects due to VPA had been reported, RCTs of pregnancy exposure were considered unethical. In human teratology research the gold standard is rigorous prospective observational studies with adequate controls over likely confounding variables. There have been reservations about accepting the results of some studies in this area because of poor study design including small study numbers, lack of blinding of assessors, presence of many confounding factors and biased ascertainment [33]. Fortunately, although we needed to rely on lower levels of evidence for some recommendations, there have been several large prospectively ascertained studies of congenital malformations and neurodevelopment in this area which have replicated findings from the poorer quality studies. The findings from pregnancy registers, though subject to some bias if registers were based on self-selection, was also a source of information which contributed greatly to our understanding in this area [34]. Overall we felt fairly confident about total evidence from these diverse sources, as many of the findings were replicated across studies. There has been very little data on medical follow-up and health surveillance in this population [35]. Much of the information available in this area has been driven by lay support groups [27]. The anecdotal case report data was considered fairly weak, as many cases were reported many years ago, before the advent of tests which could exclude genetic disorders, and some cases were exposed to polytherapy or were subject to other confounders. One relevant point is that the working group which was convened included a diverse range of professionals (Clinical Geneticists, Paediatricians, Teratologists, Neonatologists, Clinical Psychologists, Child Psychiatrists, Paediatric Surgeons and others) who had a wealth of professional experience in the diagnosis and management of the health and clinical follow up of individuals with FVSD. That said, in light of the lack of systematic evidence pertaining to health and clinical follow up, consideration of this area is likely subject to certain biases.

The articles were initially reviewed by JCS, RB and MR and from these 8 core articles [6, 10, 12, 19, 22, 27, 36, 37] were selected based on a) the level of evidence they provided ranging from 1 metanalyses/systematic reviews or RCTs with low risk of bias, through to 4, expert opinion, b) the size and design of the studies c) their relevance to diagnosis and management of FVSD. Where possible, papers with obvious bias (small studies, retrospective studies, un-blinded studies, ascertainment of participants from biased groups) were avoided, but due to the lack of papers with a high level of evidence for certain aspects of the condition, and the fact that we were interested particularly in aspects of clinical management (which is under researched), we decided to include some retrospective cohort studies if they were larger or provided information about aspects of FVSD not otherwise documented elsewhere. We took into account the bias that inclusion of these papers might introduce. The core papers selected included two Cochrane reviews on congenital malformations and neurodevelopment [19, 22]. The eight core articles, together with the translated PNDS document [30], the list of congenital malformations from case reports referred to above and a paper unpublished at the time but now in print, documenting the cognitive and neurodevelopmental profile in Fetal Valproate Syndrome [37] were graded for level of evidence and sent to each expert in the group for review. In addition, for each of the areas (e.g. cardiology, ophthalmology, neurodevelopment), the expert was also provided with all available specific papers pertaining to his/her topic. We asked each member to make management recommendations based on the evidence provided, and to provide further evidence and references from their own field if additional recommendations, not covered in the literature provided, were considered necessary. We sought opinions from experts covering a broad number of different areas consistent with the symptomatology of FVSD, and also included a parent expert from a National Support Group within our consensus group. Each expert was requested to enter their recommendations into an Excel spreadsheet and these were then collated and discussed at an expert consensus meeting held in Manchester in March 2018. Where experts could not attend, they were asked to provide the Excel summary sheet and the rationale for their recommendations which were presented on their behalf. One participant (CD) did not complete the summary sheet but read the background literature and made recommendations which were recorded by JCS at a face to face discussion and presented at the consensus meeting.

The day long consensus meeting was attended by 16 members of the consensus group. The proposed recommendations were presented and debated. During the discussion we also debated additional sources of data known to individual experts. We made use, for example, of unpublished data from follow-up to age 6 of a prospective cohort of UK children in Liverpool and Manchester exposed to antiepileptic drugs in pregnancy in whom physical examinations and measurement of growth parameters had been undertaken at age three and 6 years, classing it as lower level evidence in the light of the fact that it remains unpublished. Where there were differences of opinion these were debated and draft recommendations re-worded until they were acceptable to the majority of the group. When making recommendations we took into account the applicability of the available published evidence to the population we were targeting, how consistent the body of evidence was, how feasible it would be to tailor the recommendations to health and education systems across different countries, and the balance of benefits and harms of the recommendations made.

Following the expert consensus meeting a draft list of all of the recommendations was drawn up and circulated to members of the group who were asked to score these. Table 2 outlines the process for scoring the recommendations. Members were asked to indicate whether they agreed or disagreed with the recommendation and, if disagreeing, to state the reason. They were allowed to abstain from scoring the recommendation if it was outside their area of expertise. They were also asked to comment as to whether the recommendation was, in their opinion, based on evidence and/or consensus agreement, or covered an area where there was agreed best practice. Recommendations scored as C after this process were discarded or re-worded and re-scored until acceptable as A or B. Only recommendations scoring A or B were ultimately kept.

**Table 2:** Process For Agreeing and Scoring Recommendations

Agreement Score	
+++	> 75% agree with the recommendation
++	50–74% agree with the recommendation
+	25–49% agree with the recommendation
–	< 25% agree with recommendation
Evidence Score	
A	Based on evidence +/− expert consensus
B	Based only on consensus agreement and/or best practice
C	No evidence or consensus agreement/not currently specified as best practice

After collating the scores, a first draft of the consensus statement was circulated to all members of the expert group. It was revised based on their feedback and then circulated to a broader group of experts and patients/parents with experience of diagnosis and management of FVSD for review and comment. This group included clinical geneticists from within the European Reference Network for Congenital Malformations and Intellectual Disability, ERN ITHACA. We also sought comments from the ERN e-PAG group (European Patient Advocacy Group) and relevant lay support groups. Evaluators were asked if they would read and comment on the draft and feedback on the general content, and the recommendations in their specific area, using a structured proforma which included questions on the language used, length and format as well as a specific request to comment on recommendations made in their field of practice and a free text box. They were requested to complete this task within 2 weeks. The consensus document was revised and following this process and a final version agreed. As supplementary information to the overview document, it had been agreed at the consensus meeting to include a number of shorter summary sheets summarising the important recommendations which are relevant to different professionals and to parents. These included a summary sheet for patients and families, one for paediatricians, one for family doctors caring for an adult with FVSD, one for educators and one for psychologists. These are included here as Additional files 1, 2, 3, 4 and 5.

Including experts with varying areas of expertise and from different countries, along with parent and lay group representation, enriched the process of care plan development and was also an educational process for those involved in the working group. Outlining the methodology of the process and the requirements from the individual experts together with provision of the overview key references to all members, clear instruction on grading of evidence and recommendations, and the availability of administrative help from a coordinator, facilitated the engagement of experts and improved the ease and quality of the process.

## Recommendations

In making these recommendations we have tried to be as explicit as possible. However, due to the lack of a body of evidence uncertainty remains in some areas and specificity was not always possible. For each recommendation we have stated 1) the recommendation, 2) to whom/the timing at which it applies, 3) the rationale for the recommendation, 4) the consensus score, 5) the level of evidence and 6) any relevant references. Where there were different options we have made this clear. We have summarised key information contributing to these decisions in the text. We have taken the key recommendations as they apply to our different target groups and formulated these into one-page summary sheets for the relevant professionals and for parents, included here as an additional file.

### Comment on measures to avoid valproate exposure in pregnancy

The group considered it important to include in this consensus statement the current guidance which has recently been put in place to ensure avoidance of VPA exposure during pregnancy. The European Medicines Agency (EMA) last issued guidance on the 23rd April 2018 [2] and this is summarised in Table 3.

**Table 3:** Measures to Avoid VPA Exposure During Pregnancy; Summary of EMA guidance from the Pharmacovigilance Risk Assessment Committee

1	VPA must not be used for treatment of migraine or bipolar disorder during pregnancy
2	VPA must not be used to treat epilepsy during pregnancy unless there is no other effective treatment available. Women requiring VPA treatment should be supported and counselled
3	VPA should not be prescribed to any girl or woman able to have children unless she is on a Pregnancy Prevention Programme. This will include an assessment of potential to become pregnant, pregnancy tests before starting and during treatment, counselling about the risks of VPA treatment and the need for effective contraception whilst on treatment, review of treatment by a specialist at least annually and signing of a risk acknowledgement form
4	Women and girls who have been prescribed valproate should not stop taking their medicine without consulting their doctor as doing so could result in harm to themselves or to an unborn child.

### Diagnosis of Fetal Valproate Spectrum Disorder (FVSD)

Bjerkedal et al. [38], who reported the results of an epidemiological study in the Rhône-Alpes region of France in 1982, were the first to draw attention to the increased risk of spina bifida after VPA exposure in pregnancy. Reports of other malformations followed, and in 1984 the first series of seven children affected by the Fetal Valproate Syndrome (FVS) was published by Di Liberti et al. [6] Further reports characterised FVS as a consistent pattern of major and minor malformations, facial dysmorphism and impaired development [8, 10, 12, 36, 39, 40]. Risks are increased in particular for neural tube defects [7, 13], congenital heart disease, cleft palate [41], radial ray defects [42–46], ophthalmological [47–49] and genitourinary anomalies [6, 50–52]. Associated minor anomalies include inguinal hernia [53], overlapping toes [12] and scalp defects [6, 54]. The facial dysmorphism is characterised by a broad nasal bridge, short nose with forward-facing (anteverted) nostrils, small mouth with thin upper lip, everted lower lip flat philtrum, ridging of the metopic suture and neatly arched eyebrows [6, 9, 10, 12, 51, 53, 55]. Problems with impaired cognition and neurodevelopment, including an increased risk for attention deficit hyperactivity disorder (ADHD) and autism spectrum disorder (ASD), went unrecognised initially but are now proven to be part of the FVS phenotype [21, 22, 24–26, 37, 56, 57].

The fact that the prevalence of the cognitive and neuropsychological deficits after VPA exposure is higher than that of the prevalence of VPA associated malformations suggests that an individual can demonstrate adverse effects of VPA exposure without necessarily having all of the physical features seen in FVS. In fact, impairment of functioning is known to occur at lower doses and at increased frequency than structural malformations across teratogenic exposures [55]. Further, studies have specifically excluded VPA exposed children with major congenital malformations and still find the increased risk of both reduced IQ [57, 58] and autistic spectrum disorder [25]. Kini et al. [59] raised the question of whether it was possible to have impaired development as a result of VPA exposure in the absence of dysmorphic facial features. The dysmorphic features associated with VPA exposure can be subtle and age dependent and designating individuals as having the characteristic dysmorphism or not is difficult, especially for those with limited expertise in the area. There are many varied presentations following exposure in utero to VPA as not every affected individual will have identical features. Due to these points the expert consensus group felt that whilst the presence of a typical facial presentation makes the diagnosis more certain, typical facial features are not absolutely required to be able to diagnose that an individual has been affected by exposure to VPA in utero, particularly if the dose of exposure was low or if the exposure occurred after the period of fetal facial development. These points provided the rationale for moving towards using the term Fetal Valproate Spectrum Disorder, FVSD, a situation akin to that used when discussing adverse effects of exposure to alcohol in utero [60]. This seems appropriate, since children with neurodevelopmental effects of VPA exposure, but without significant malformations, can be as impaired in their everyday functioning as children with classical FVS, and need to be identified in order to be offered the appropriate management.

The diagnosis of FVSD is difficult, as there is no specific biomarker that can be assayed in this condition to prove diagnosis, though there are conditions with overlapping features which need to be excluded [51]. Diagnostic criteria for Fetal Anticonvulsant Syndrome were drawn up by Dean et al. in 2000 [53] and our expert consensus group reviewed these prior to developing new criteria for FVSD which reflect our current knowledge and the now established evidence base. The revised criteria presented here in Table 4 have been divided into “essential criteria”, defined as those which must be present for an FVSD diagnosis, “suggestive” features which are seen at significantly increased frequency (> 10%) in FVSD, and “supportive” features which occur independently within the general population but are more common in FVSD. The supportive criteria are weighted according to their frequency in the general population (the more common they are in the general population the less weight they are given).

**Table 4:** Diagnostic Criteria For Fetal Valproate Spectrum Disorder. For diagnostic criteria to be met all essential criteria must be fulfilled in addition to two suggestive criteria or one suggestive plus a supportive score of 3 or more

Grade	Criterion	Comments	
Essential	Confirmed exposure to VPA during pregnancy	Any dose or duration	
Essential	Has no other recognisable diagnosis which would explain the phenotype	As evidenced on assessment by a clinical geneticist or other professional with relevant expertise	
Essential	Normal microarray-CGH and Fragile X studies	Part of diagnostic work-up	
Essential	Other teratogenic disorders with clinical overlap excluded	In particular fetal alcohol syndrome / spectrum disorder	
Suggestive	Facial dysmorphism consistent with VPA exposure (flat philtrum, thin upper lip, full, everted lower lip, short anteverted nose, small mouth, epicanthic folds, neat arched eyebrows, broad nasal root) see Fig. 3	Include review of photographs at a younger age and take into account variability of phenotype with age (see Fig. 3)	
Suggestive	Cognitive profile consistent with current knowledge of that associated with valproate exposure	a) discordant from parents b) in infancy: motor and speech delay, c) school aged: IQ, verbal reasoning, communication and executive functioning deficits	
Suggestive	Presence of social communication difficulties/autism spectrum disorder	Occurs in 6–15%	
Suggestive	Spina bifida	20 fold risk	
			Score
Supportive	Congenital cardiac defect	Confirmed on echo	2
Suggestive	Cleft palate		2
Supportive	Metopic suture synostosis		2
Supportive	Radial ray defect	Includes mild variants with flat thenar eminences	2
Supportive	Genitourinary malformations	Hypospadias, abnormal collecting system, hydronephrosis	2
Supportive	Laryngomalacia/stridor		2
Supportive	Joint laxity	Beighton 6 or more	1
Supportive	Talipes requiring surgery		1
Supportive	Digital anomalies	Overlapping toes, camptodactyly, clinodactyly	1
Supportive	Ophthalmological anomalies	Coloboma, strabismus, refractive error	1
Supportive	Enuresis/poor bladder control	Requiring investigation	1

**Fig. 2:**
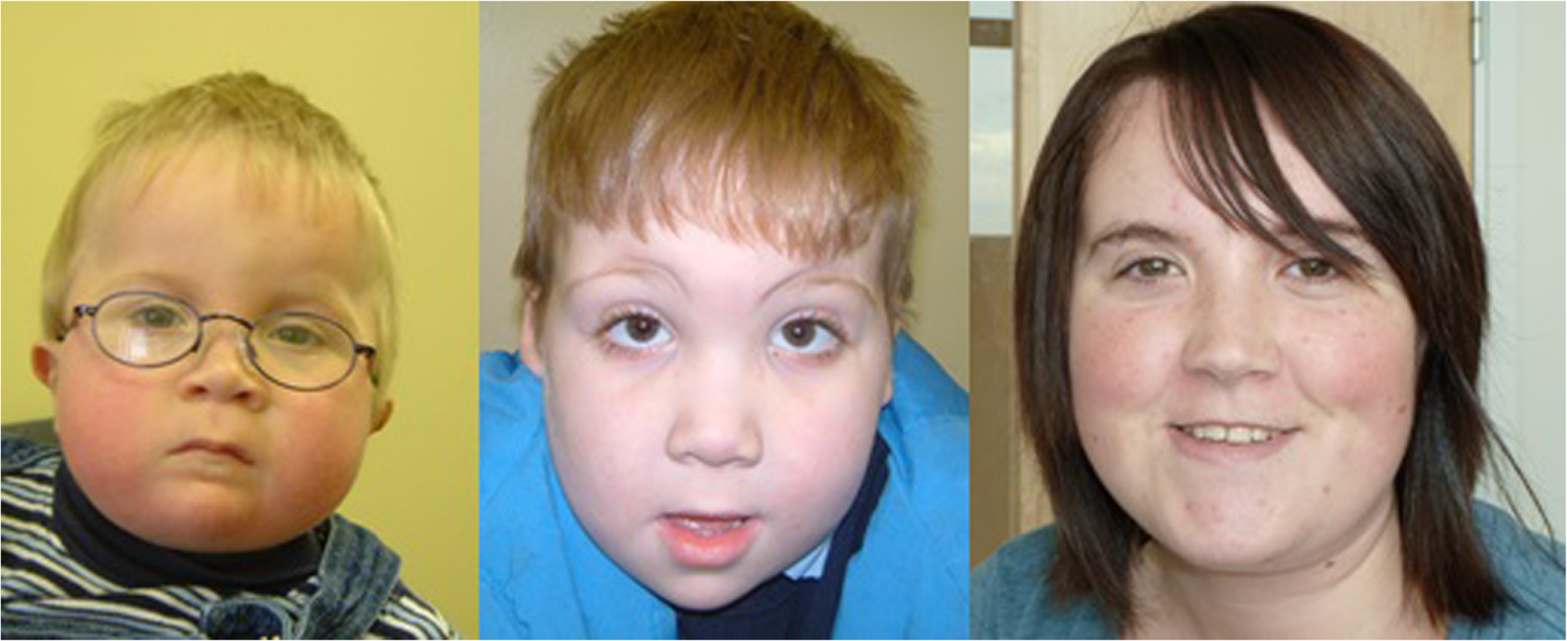
Facial Features Associated With Valproate Exposure At Different Ages. Note the presence of anteverted nares, small mouth, thin upper lip, everted lower lip, flattening of philtrum, prominent midline to forehead. Features are attenuated but still apparent in young adult

**Fig. 3:**
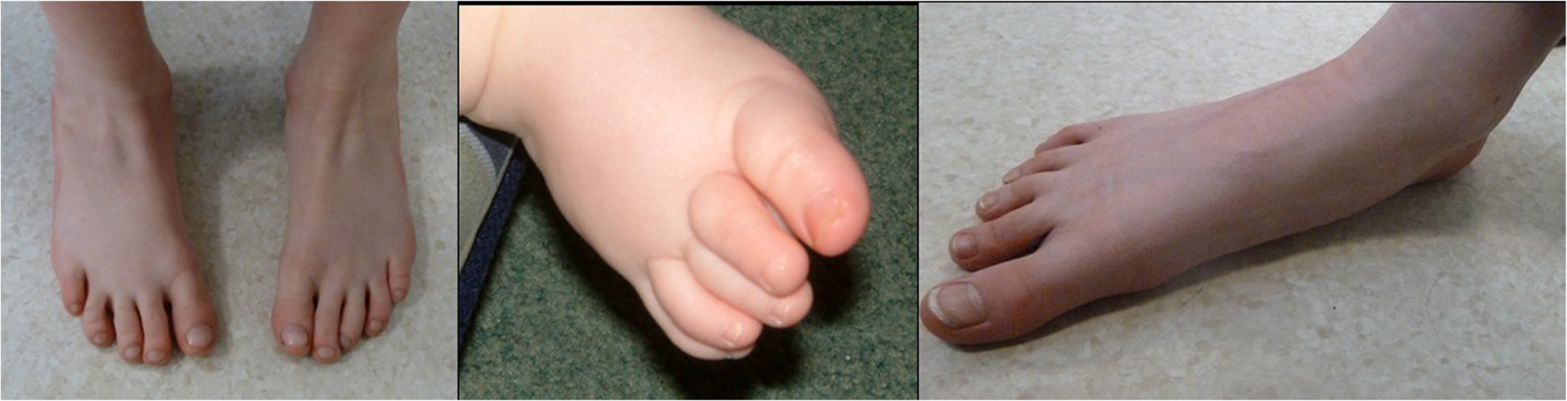
Minor limb malformations Seen After VPA exposure. Note the hypoplastic and overlapping toes and flattening of the arches due to the joint laxity frequently seen in FVS

Dysmorphic facial features have previously been considered as a strong diagnostic handle for FVSD [59] as these appear specific to the condition. Figure 2 demonstrates the classical facial features, which change over time but are still recognisable in adults. In view of the fact that they may be difficult to recognise in their milder form by those who are not expert in the area, and that there is debate as to whether their presence is required, they have been listed as “suggestive” rather than “essential” criteria. Figure 3 shows limb malformations which are associated with valproate exposure.

Given the prominence of the cognitive, social and motor difficulties within the presentation of FVSD, individuals with the condition should be referred to a clinical psychologist or neuropsychologist as part of the diagnostic process for objective assessment of cognitive and neuropsychological problems unless the child clearly has a severe neurodevelopmental impairment in which case the presence of this will be clear. The focus of the assessments will vary by age but should cover cognitive, speech and motor development in infancy and IQ, language, memory, attention and executive functioning at school age or older.

## Management of Fetal Valproate Spectrum Disorder

In view of the fact that multiple body systems are affected in FVSD the need for a multidisciplinary model of care was acknowledged and strongly supported by the group. As the signs and symptoms vary with age we felt it important to encompass preconception and prenatal management as well as management of the neonate, child, and adult. The recommendations for management have been divided according to the clinical setting and age at which individuals are seen rather than by body system, and are listed in Table 5. Where literature evidence is available references are given and papers listed at the end of the document.

**Table 5:** Recommendations For The Management Of Fetal Valproate Spectrum Disorder

Recommendation	Agree +++ > 75% ++ 51–74% + < 50%	Evidence Strength A B C
Diagnosis		
Diagnosis should be made by a clinician with expertise in the area based on the stated diagnostic criteria	+++	B
Microarray analysis and Fragile X studies should be undertaken and other syndromic diagnoses suggested by history and clinical findings ruled out [53]	+++	A
The diagnostic process should include a developmental/neuropsychological assessment [53]	++	A
Preconception Counselling		
Treatment with VPA should be supervised by a doctor specialising in the area who will offer advice according to EMA and country-specific guidance [2, 61]	+++	A
Women and girls taking VPA who find themselves pregnant should not stop taking their medication without consulting their doctor [61]	+++	A
Possible genetic causes for the mother’s seizures should be considered and investigated if appropriate [62]	+++	A
Recurrence risks for seizures in a child should be assessed and communicated [62]	++	B
As for all pregnancies, folic acid should be taken from 2 to 3 months before conception and continued until 12 weeks [63]. The dose to be taken varies between countries. After the first trimester doses high doses are not recommended or justified [64–66]	++	A
Pregnancy and Neonatal Period		
In pregnancies where VPA is being taken women should seen in a joint obstetric/neurology clinic [63]	++	B
Ultrasound scans should be carried out at around 13 and 20 weeks depending on country. Additional scans are not needed if the heart can be visualised adequately at 20 weeks	++	B
The ultrasonographer/radiologist should be made aware of a history of VPA exposure and the type of structural anomalies which are associated and can be looked for especially on scan	+++	B
If a major fetal malformation is detected on antenatal scans, discussion as to the most appropriate place for delivery should take place after detection of the anomaly [63]	+++	B
The neonatal check [67] should be carried out by a professional with knowledge of FVSD. Special care should be taken to visualise the palate directly and to check for submucous cleft palate, to look for radial ray defects, talipes and ridging of the metopic suture indicating metopic synostosis.	++	B
If spinal dysraphism is suspected clinically e.g. the presence of dermal sinus, a skin dimple which is very large (> 5 mm) or high on the back or if there is ultrasound evidence of a spinal cord anomaly, the child should be referred to a paediatric neurosurgeon for clinical assessment and further imaging. Small sacral skin dimples/pits can be safely ignored [68, 69].	+	B
Midwifery/paediatric staff should be alert for signs of hypoglycaemia and blood sugar should be checked if these occur [70].	+++	B
Breast feeding should be encouraged [71, 72]. Feeding should be established before discharge home	+++	A
Airway symptoms should prompt assessment by a suitably experienced airway surgeon [73]. Assessment may include rigid airway endoscopy under GA to exclude laryngeal abnormalities and tracheomalacia.	+++	B
If signs of drug withdrawal are present these should be treated [70, 74]	+++	A
If talipes is present referral should be made to a physiotherapist/or paediatric orthopaedic surgeon as required [75]	+++	B
Before discharge, arrangements for follow-up of the baby with a paediatrician and/or other specialists should be put in place. This professional will be responsible for coordination of health surveillance and care	+++	B
A renal ultrasound should be arranged after birth for all VPA exposed infants [18, 19]	+++	A
A one-off cardiac echocardiogram should be arranged [18, 19]	+++	B
An epilepsy nurse should see the mother after delivery to review her seizures and medication and discuss childcare and contraception.	++	B
Paediatric Surveillance: General Points		
At all ages, if malformations, medical problems or developmental problems are detected the paediatrician should refer the child on to the appropriate specialist	+++	B
Height, weight and head size should be measured at each paediatric visit and plotted on the appropriate growth charts	+++	B
Enquiry about hearing and vision problems should be made at each visit [27, 47]	+++	B
If developmental problems are suspected at any stage referral should be made for a comprehensive developmental assessment	+++	A
Paediatric Assessment and Health Surveillance of Infants		
In addition to the routine health checks by the health visitor, a paediatric appointment should be arranged at the age of six to eight weeks and further review at 9 months	++	B
A full examination should be carried out in the first year to detect malformations. This should include detailed examination of the hands and forearms to look for signs of radial ray defect. Minor signs of this may include a hypoplastic thumb or flat thenar eminence	++	B
Hip stability should be checked and ultrasound scan arranged if any concerns	++	B
Where talipes is resistant to treatment or where there are abnormal neurological signs in the legs imaging of the spine should be carried out [69, 75]	+++	A
If there are clinical concerns about head shape or growth the child should be promptly referred to a Craniofacial Unit for evaluation and possible treatment [76, 77]	+++	B
A developmental history should be taken and development should be assessed	+++	B
An ophthalmological examination including orthoptic assessment, full ocular examination (to look for iris and chorioretinal coloboma, cataract and other abnormalities), and cycloplegic refraction should be carried out during the first 6 months of life [78]	+++	B
Early intervention for hearing loss is indicated to limit impact on development	+++	B
Paediatric Surveillance At 18 months		
Health and development should be reviewed by a paediatrician or an appropriately trained health professional at 18 months	+++	B
A screen for autism spectrum disorder/social communication disorder should be undertaken by the community paediatrician	+++	B
Surveillance During Childhood		
After the 18 month check progress should be reviewed by an appropriate health professional on an annual basis until school age, with a check being carried out prior to starting school. Thereafter appointments should be arranged the year before leaving junior school (age 10) and the year before public exams (age 13–14).	++	B
If there are concerns about speech and language a referral for formal assessment by a speech and language therapist should be made by 2.5 years	++	B
Joint hypermobility should be considered as a cause in those presenting with leg pains and assessed by Beighton score [79]. If score > 6 referral to a paediatric physio should be made and an age appropriate graded exercise programme administered to build up core muscle strength and endurance. This might also include swimming and soft play. Special requirements for joint hypermobility should be included in the individualised educational plan.	+++	B
Offer referral to a physiotherapist for fatigue management and advice on management of joint laxity and posture where these are prominent features	+++	B
Where there are foot deformities or flat feet referral to a podiatrist for orthotics should be made [80]	+++	A
Good posture and spinal strengthening exercises should be recommended	++	B
Enquiry about sleep disordered breathing should be made at each surveillance visit	+++	B
Children with impaired hand function should be referred to an occupational therapist	+++	B
An ergonomic assessment of the school environment should be undertaken if required by an occupational therapist to ensure that the environment is suited to any specific needs of the child	++	B
An annual ophthalmic assessment to look for amblyopia, strabismus, refractive error and other abnormalities should be carried out. Spectacles should be prescribed as appropriate, and treatment for amblyopia and strabismus carried out as required.	+++	B
Regular hearing assessment, including otoscopy should be carried out on all children and adolescents with FVSD	++	B
Specific enquiry should be made about symptoms of urinary tract infection and enuresis. If present these should be managed in the standard way	+++	B
Development should be assessed and enquiry should be made about school progress at each visit	+++	B
Where developmental problems are identified a referral to a psychologist should be made so that appropriate specialist advice can be given to schools	+++	B
By virtue of having a mother with epilepsy children with FVSD may be at increased risk of seizures. If these occur they should be managed in the standard way. Genetic testing for seizure predisposition genes should be considered [62]	+++	B
Children with objective cognitive impairment should have an individualised educational plan or equivalent drawn up and be offered tailored input with their education	+++	B
Educators of children with FVSD should be provided with information about the condition and about strategies to best support affected individuals	++	B
Management of Adolescents With FVSD		
The need for extra time or a scribe should be assessed when taking examinations	++	B
Health and social care service managers in children’s and adults’ services should work together in an integrated way to ensure a smooth and gradual transition from paediatric to adult care and individuals with FVSD should be involved in the transitional arrangements wherever possible.	+++	A
Annual assessment by an optometrist for refractive error is recommended	+++	B
Management of Adults With FVSD		
A designated general practitioner should be identified to coordinate care	++	B
An individualised educational plan should remain in place with annual reviews as long as it is necessary. In some countries this is also allowable for adults	+++	B
An annual health check for medical problems in learning disabled patients should be carried out by the general practitioner [81]	++	B
Weight should be monitored at the annual check and dietary advice given if overweight for height	+++	B
Referral to a podiatrist should be undertaken where foot posture remains abnormal	+++	B
An occupational therapy referral should be made so that an ergonomic assessment of the workplace or educational setting should be carried out and adaptations made for any special needs	+++	B
Offer referral to a physiotherapist for fatigue management and advice on management of joint laxity and posture	+++	B
Annual assessment by an optometrist for refractive error should be recommended	+++	B
Anaesthetic Management Of The Individual with FVSD		
Preoperative evaluation should include a full assessment of potential risk factors including a cardiological assessment and ENT evaluation of the upper airway.	+++	B
Dysmorphic features such as micrognathia, small mouth and cleft palate should be assessed as they can alert the anaesthetist to a potentially difficult airway and difficult endotracheal intubation	+++	B
Anaesthetists should have a high index of suspicion to subglottic narrowing and ENT presence may be required to examine the lower airway in full.	+++	B
Postoperative care will require monitoring for apnoeas in neonates and infants.	+++	B

A Supported by evidence from literature or full consensus

B Considered best practice in the area but lack of specific evidence

### Preconception care and advice

Preconception care in women with epilepsy has recently been the subject of European debate, with some countries also issuing their own guidance [61, 82, 83]. In April 2018 the European Medicines Agency endorsed new measures to avoid valproate exposure in pregnancy [2]. Essentially, VPA may not be used to treat bipolar disorder or migraine in pregnancy and can only be used for the treatment of epilepsy if women are on a pregnancy prevention programme and sign documentation affirming that a discussion of the risks has taken place. It is recommended that folic acid, taken in doses 0.4-5 mg daily should be taken from 2 to 3 months before conception and should be continued until 12 weeks of pregnancy. Recommendations of the dose vary widely between countries and societies. The Medicines and Healthcare Regulatory Agency (MHRA) recently issued specific guidance to all women for whom VPA is being prescribed [82]. A daily dose of 4-5 mg is recommended if there is a family history of neural tube defect. The evidence of high dose folic acid in preventing VPA teratogenesis is conflicting [64, 65]. A recent small trial of whole pregnancy folate supplementation suggested that there are psychological developmental benefits for children [84]. However, current evidence suggests that after the first trimester, high doses are not recommended or justified [66, 85, 86].

### Pregnancy

The risk of congenital malformations in babies exposed to VPA in pregnancy is of the order of 10–11% but increases as the dose increases and can be as high as 24% [87]. Some malformations are specifically increased after VPA exposure. The European Surveillance of Congenital Anomalies (EUROCAT) antiepileptic study database, gathering data from several congenital malformation registries [88], suggested a relative risk for neural tube defects of 12.7, whereas Weston et al. in the Cochrane review on malformations [19] identified a lower relative risk of around 5.3 from prospective observational studies. The risk of cardiac malformations is increased 2–3 fold, that for cleft palate increased five-fold, that for craniosynostosis six-fold, and that for hypospadias and genitourinary (GU) malformations five-fold. Limb malformations, more specifically radial ray defects, are also increased. The effects are dose related [88]. In view of this increased risk malformations should be specifically sought on a routine detailed anomaly scan at 20 weeks and the fetal medicine specialist or ultrasonographer carrying out the scan should be made aware of the history of VPA exposure. However, although it is possible to visualise many of the structures concerned, cleft palate and some heart defects may go undetected. Contrary to early literature, cleft lip is not increased in frequency [41]. Further scans are not necessary if the 20 week scan is normal. If abnormalities are detected on the fetal anomaly scan these should be discussed with the parents and may require follow-up scans. In the event that a woman becomes pregnant unexpectedly whilst taking VPA she should not stop her medication without consultation with her neurologist, as uncontrolled seizures pose a significant risk to her and possibly, although less clear, the unborn child. The incidence of intrauterine growth retardation and Caesarean section is not significantly increased in mothers taking VPA in pregnancy [52]. Where a major malformation has been identified during pregnancy, and the pregnancy continues, delivery should be planned to take place in a hospital with the appropriate facilities for neonatal care and the neonatal team should be involved during the pregnancy.

### Neonatal period

The majority of infants exposed to VPA during pregnancy will have a normal neonatal period [52]. Though the NEAD study [89] did not find any difference in Apgar scores across infants exposed to different AED monotherapy drug types, two studies [90, 91] have found a near doubling of risk of having a lower Apgar score in VPA exposed infants in larger cohorts. There have been some reports of neonatal withdrawal symptoms and hypoglycaemia. Though Thisted et al. [70] observed these in 13/22 VPA exposed infants, in a subsequent prospective study of 227 women with epilepsy (WWE) and 315 control women [52], there was no significant difference in neonatal problems or admission to the neonatal intensive care unit between the two groups. In a study from Norway, in which 215 babies were exposed to VPA, there was no increased incidence of neonatal hypoglycaemia [74]. VPA is not an enzyme-inducing AED and so the risk of haemorrhagic disease of the newborn is not increased unless mothers are taking other enzyme-inducing medications. Oral administration of Vitamin K to mothers during pregnancy is not specifically needed [63]. In many countries however, 1 mg Vitamin K given intramuscularly is offered to all infants after birth.

VPA does not pass through into breast milk in large concentrations [92]. A study by Meador et al. of the IQ of children exposed to VPA who were breast fed compared to those who were not demonstrated no adverse effects of breastfeeding and a higher overall IQ for breastfed infants [93]. A further Norwegian study failed to demonstrate an adverse impact on neurodevelopment at 6 and 18 months [71]. Whilst further evidence is required, currently breastfeeding should be encouraged [72]. A thorough neonatal baby check [67] is essential after birth because of the higher risk of both major and minor malformations. Particular effort should be made to visualise the palate, to check for limb defects, most common involving the radial rays [42–46], to check the spine, and to note any dysmorphic facial features (Listed in Table 4) which are often very recognisable in the neonate. Pronounced ridging of the metopic suture with trigonocephaly [12, 76, 77] may indicate craniosynostosis which will require a referral to a craniofacial team. The risk for malformations of the genitourinary (GU) tract and the heart is increased. Though septal cardiac defects are more common, in some instances very complex congenital heart defects, which may be difficult to detect in neonates, may be present [94] and so one-off scans of the renal tract and the heart should be arranged after birth. Infants exposed to VPA have a significantly increased risk for neural tube defects, most of which will be detected in utero. The occurrence of occult neural tube defects after VPA exposure in humans has not been well documented in the literature but is seen in animal models [95], therefore, checking for signs of occult lesions such as large (> 5 mm) sacral dimples or dermal sinuses is recommended. Small sacral dimples and pits can be ignored[68]. A further associated malformation is abdominal wall defect (omphalocele) [10, 12, 96], which is obvious after birth and will need immediate surgical intervention. Airway problems, principally stridor due to laryngo or tracheomalacia, are increased in frequency [10, 12, 39, 40] Infants with these problems should be referred to a specialist in airway management for assessment. Common minor malformations which may be noted include overlapping or hypoplastic toes, especially the fifth toes [12], minor differences in the ear shape and postural talipes deformity [12, 53]. These usually become less obvious over time and resolve with minor intervention.

Following delivery, it is recommended that the mother should have a chance to meet with an epilepsy specialist nurse who will review her seizure management and provide practical advice on how mothers with epilepsy can safely care for their baby once discharged home. At the time of discharge the first follow-up appointment with the paediatrician should be arranged for the baby and the local midwife and health visitor or equivalent should be made aware of the need for extra surveillance.

### Paediatric surveillance of the infant

It is recommended that children exposed to VPA in utero should undergo a number of checks during childhood, timed to fit in with routine health checks and specific developmental stages. At each of these, growth, development, hearing and vision should be checked and any new problems identified, and referrals made to specialists as appropriate. In addition to the normal baby health checks a paediatrician should review the baby at 6–8 weeks of age. This provides an opportunity to look for any malformations which may have become apparent since birth and check that the necessary screening investigations have been arranged. Some malformations such as minor radial ray defects, for example, may have been difficult to assess straight after birth. A referral can also be made at this stage to an ophthalmologist so that screening for ocular malformations, especially retinal coloboma, can be carried out. This visit also provides an opportunity to check for hip stability as the joint hypermobility seen with FVSD [27] can be a contributing factor to congenital dislocation of the hip [14].

### Pre-school surveillance

In addition to routine toddler health checks a review by a paediatrician is recommended at eighteen months of age as this is a key time for language assessment and screening for symptoms of social communication disorder. Thereafter, annual health checks should be carried out until school age by a community paediatrician or appropriately trained specialist nurse with monitoring of growth and enquiry about development, hearing and eyesight problems being undertaken at each visit. These visits provide an opportunity to check for symptoms known to occur with increased frequency in FVSD. Evidence of neurodevelopmental difficulties merits referral for neuropsychological evaluation prior to or during the first year of school to ensure adequate educational support and placement. It should be noted that children may ‘grow into’ certain cognitive, social and developmental deficits and for this reason a single follow up in the infant years is not adequate [97].

### School age surveillance

The aim of surveillance during the school years is to make sure that any problems caused by VPA exposure are recognised and managed appropriately during the period of the child’s education, whilst not incurring too much time off school for hospital appointments. This ensures efficient use of healthcare resources, parents are not excessively inconvenienced, and children are not marked out as different or disadvantaged academically because they need repeated days off school. The consensus group therefore agreed on reviews taking place in the school year prior to the move to high/secondary school and the school year prior to taking public examinations and making career choices. The age for these might vary slightly from country to country. As with other paediatric checks, enquiry about any medical problems, hearing or eyesight problems, and difficulties with school progress, behaviour and social interaction, should be made, with referrals made to appropriate specialists (i.e. psychology or psychiatry) where necessary. At 16–17 years, arrangements should be made for transition to adult care, the latter usually being undertaken by the family doctor. At this point the doctor taking on annual health checks should be provided with details of past history and screening investigations, a summary of any current concerns and a checklist such as the one included as Additional file 3 outlining the ideal management plan during adulthood.

Recommendations should be provided to schools when required. Individuals with FVSD should be referred for a neuropsychological assessment, or for a comprehensive cognitive assessment which includes, but is not limited to, IQ, memory, language, executive and attention abilities. This will allow recommendations to the school and the family to be tailored., creating a more bespoke intervention. As a minimum, a high proportion of individuals with FVSD are likely to require additional support in school around the learning of new information and when children are required to take examinations, an assessment should be made as to whether extra time or a scribe is needed.

The risk of developmental delay after exposure to VPA in utero has been reported to be in the region of 30% [98–104], but the risk of adverse cognitive and other neurodevelopmental outcomes is far greater when there are physical signs of VPA embryopathy [27, 28]. Effects on development have been seen in the absence of congenital malformations [58, 101]. They are dose-related [103], so children exposed to higher doses of VPA are at greater risk. In a Cochrane systematic review of neurodevelopment [22], Bromley et al. concluded that there was an average reduction of 9 points in the developmental quotient between children exposed to VPA and unexposed or control children, with the risk to school aged IQ being a 7–11 point reduction in comparison to both controls and other AED exposed children. Lowering of group mean scores leads to an increase in the number of children falling below the average range. For IQ, prospective studies suggest that around 20–30% of individuals with FVSD will demonstrate below average IQ; however declines in ability are very much dose dependent [22]. In addition, further studies have shown impairment of memory [37] and Erikkson et al. reported more difficulty in remembering faces and learning lists [101]. From a practical point of view, Adab et al. [98] in a retrospective case control study demonstrated that VPA exposed children had a 3.4 fold increased risk for being assessed as having special educational needs and the prospective study by Baker et al. [21] calculated that this risk was even higher, ie around eight fold, if exposure was to high dose VPA (defined as doses above 800 mg daily). Elkjaer et al. [104] found that a population of Danish schoolchildren had lower grades in Danish and maths if they had been exposed to VPA. The observations, overall, translate into a high number of VPA exposed children requiring additional input with their education, especially if exposed to doses of VPA greater than 800 mg daily [105]. A recent population-based cohort study from Denmark by Christensen et al. [106] suggested that there is a 48% increased risk of having attention-deficit hyperactivity disorder (ADHD) after prenatal exposure to VPA. In addition to the documented cognitive and academic difficulties, children with FVSD may have problems with organisational and social skills which impact on their school progress. Parents have identified that training of educators including teachers and teaching assistants to inform them about the spectrum of difficulties seen in FVSD and strategies which can be adopted at school to overcome these is of significant benefit. As for all children with disabilities, adjustments to classrooms or school buildings may also be needed. In the UK this is covered under the Equality Act 2010 and other countries have similar legislation.

### Speech and language

Prospective studies of cognitive development in children exposed to VPA during pregnancy have consistently shown a clinically significant reduction in full scale IQ compared to control populations, with greater impairment of verbal IQ. Specific impairment of language skills has also been documented [105] together with deficits in auditory working memory [56, 105]. The most notable aspect of the FVSD cognitive phenotype known to date is the frequent discrepancy often seen between verbal and non-verbal IQ, favouring non-verbal IQ [37, 56, 98]. There is both prospective and retrospective evidence that children with a history of VPA exposure are at risk of poorer language development [27, 35, 57]. In view of these observations it is recommended that all VPA-exposed children are carefully monitored for language delay. Those with language deficits on a screening assessment at 18 months of age should be referred to a speech and language therapist (SLT) for intervention if resources permit. Referral should be made to the SLT by 2.5 years at the latest. When decisions about school placements are made, it is recommended that children with FVSD should be offered a place at a school where staff have had speech and language communication needs (SLCN) training. If specific speech, language and communication needs have been confirmed, then ideally child would be offered a place at a specialist language unit attached to a mainstream school. If this is not possible, then regular assessment/reviews of progress and advice/intervention should be offered by a qualified SLT as required.

### Social communication disorder and autism Spectrum disorder

The prevalence of autism spectrum disorder (ASD) measured in a UK population is around 1% [105]. Early case reports had mentioned autism spectrum disorder in children exposed to VPA. A prospective study carried out in Manchester reported an incidence of 6.3% for ASD in children exposed to VPA monotherapy [25] and a Scottish population-based study [24] reported an incidence of 8.9% for ASD in an exposed group. Christiansen et al. [25] undertook a population based study in Denmark of over 65,000 children born between 1996 and 2006 to see if ASD was significantly increased after VPA exposure. The study identified a doubling of risk for childhood autism in the 432 VPA exposed children. Wood et al. reported their findings of a prospective evaluation of autistic traits [107]. When screening for ASD traits, the incidence of ASD was 7.7% in a group exposed to VPA monotherapy and 46.7% in those where VPA was used as polytherapy; although the group size was small. Risk of autistic spectrum disorder and social difficulties has been demonstrated to increase with increasing dose of VPA. This also ties in with the observation of an ASD-like phenotype in rodents exposed to VPA during pregnancy [107]. Clinical experience backs up the finding of Wood et al. that there are a significant number of individuals with FVSD that may not meet diagnostic criteria for an autistic spectrum disorder but have significant difficulties with social communication. Given that there are now early intervention programmes for ASD [108, 109], screening and formal assessment for this is warranted so that symptoms can be detected early, appropriate help can be put in place, and a diagnosis of ASD can be factored in when planning school placements.

### Growth and general health

Birthweight in VPA exposed children does not differ from that of control or unexposed children [8, 14, 27, 52]. There is little data on growth as few long term follow-up studies have studied this, but unpublished data from follow up of the Liverpool and Manchester Prospective cohort presented by Mawer et.al. [52] which included 57 infants exposed to VPA monotherapy and 283 controls has not identified any significant differences in height, weight or head circumference at the age of 6 years. As regards older individuals with FVSD, the consensus group made the personal observations that weight may increase from the time of puberty so this should continue to be monitored and dietary and lifestyle advice given where needed. There are some reports of early puberty but this has not been formally studied.

It should be noted that although a number of medical problems have been reported in VPA exposed children [27], for the most part their management is no different to that of other children with the same complaints. In a large population based study, the rates of GP contact were increased for valproate exposed children but the increase was small [110]. Consistently, in the Liverpool/Manchester cohort [52], 20/57 (35%) of the VPA monotherapy patients, and 12/26 (46%) exposed to VPA as polytherapy, had needed to consult a doctor for a medical problem compared to 65/283 (23%) controls (unpublished data). Similar types of problem were encountered in both groups and included asthma, eczema, upper respiratory tract infections and otitis media. Moore et al. [27] reported the presence of otitis media in 19/57 children with AED exposure, 15 of whom were exposed to VPA monotherapy (44%). This was a somewhat biased group, ascertained through a Fetal Anticonvulsant Support group, and caution is required in interpreting these findings. In the Liverpool/Manchester prospective case controlled cohort study [52] the incidence of hearing problems and otitis media did not differ between case and control cohorts (unpublished data). In fact 80% of otherwise healthy children experience otitis media before 10 years of age and 40% of 2 year olds [111] and therefore this was not deemed to be a specific symptom of FVSD by the consensus group. However, it could be hypothesised that the increased incidence of cleft palate, a risk factor for conductive hearing loss, together with subtle differences in structure of the mid face and skull, may make some within the FVSD population slightly more susceptible to recurrent episodes of otitis media. One older individual with FVS has required treatment for a cholesteatoma (personal communication regarding patient in cohort reported by Mohd Yunos et al. [112]. Surveillance for otitis media, to include otoscopy at each clinic visit seems prudent as it would be important that risk of conductive hearing loss is minimised in a child who may have other disabilities. The occurrence of hyperacusis has been reported frequently in VPA children by parents but has not been studied formally. It can, however, pose problems in noisy environments, including schools. Delayed toilet training and enuresis have been reported. In the Liverpool/Manchester study referred to above, 12/196 (6.1%) completing a health questionnaire at 6 years had functional bladder problems but so did 14/256 (5.4%) of the control cohort (unpublished data). In this same cohort 11/196 (5.6%) had a GU malformation diagnosed by the age of 6 years compared to an incidence for similar malformations of only 5/256 (1.9%) in controls. As there is an increased risk for structural GU malformations, a one-off scan of the kidneys and urinary tract is recommended after infancy. If present, renal anomalies will require management as for any child with similar malformations and prophylactic antibiotics or surgery may be needed. In later childhood and adolescents, enquiry regarding enuresis and urinary problems should be made with referral to appropriate specialists. There have been no specific studies of this problem in FVSD, but there are several anecdotal reports of impaired bladder sensation and enuresis affecting social activities e.g. participation in school residential trips and clinical experience within the consensus group suggested that functional problems of this type appear to be more common in FVSD. The majority of children with FVSD are, by virtue of having a parent with a seizure disorder, at higher risk of having seizures themselves. Seizures do not appear to be a consistent feature of VPA exposure per se, and have rarely been documented in reports, but the seizure risk would be increased if a mother passes on a dominant seizure predisposing genetic variant to a child [62]. In cases of familial epilepsy, consideration should be given to screening for underlying genetic variants in seizure predisposing genes. Some of these variants might also have an impact on intellectual development [113].

### Ocular anomalies

Glover et al. [47] documented the ocular findings in 27 individuals exposed to valproate monotherapy. 50% had myopia of > − 1 dioptre and in 28.6% the refractive error was greater than − 4 dioptres. 11% of valproate exposed children had anisometropia and strabismus was common. Myopia had gone undetected or untreated in a significant proportion of cases. Shah et al. [48] and Jackson et al. [49] both reported the occurrence of coloboma in valproate exposed infants. A blocked or absent lacrimal tear duct may also occur at increased frequency (Turnpenny, personal communication). In view of the occurrence of both congenital malformations which might impact on vision and the high risk of development of a significant refractive error, we have made recommendations for both an early ophthalmological assessment and ongoing surveillance for refractive error.

### Joint hypermobility

In the case series reported by Moore et al. [27] joint hypermobility was a prominent feature in the VPA-exposed children. It had been documented in 24/34 valproate monotherapy patients and 10/12 VPA polytherapy patients. In addition hernia was present in 4 of these. The finding of joint hypermobility in VPA exposed children has been reported frequently by others [12, 39, 40] with hypotonia also described as a feature in many cases. If present it should be managed in the standard way. A Beighton score [79] should be measured at the school age clinic visits and if the score is greater than 6 a referral to a paediatric physiotherapist should be made and an age appropriate graded exercise programme administered to build up core muscle strength and endurance. Good posture and spinal strengthening exercises are recommended. Exercises might also include swimming and soft play. Special requirements for joint hypermobility should be included in the formal education plan for the children. Hypermobility can manifest as leg pain if weight bearing when walking tends to be on the insides of the feet, and a referral for orthotics should be made. Joint hypermobility can cause problems at school in covering longer distances between classrooms and stairs, and in holding a pen to write for long periods. An ergonomic assessment of the school environment by an occupational therapist should be undertaken to highlight problem areas in those cases where there are significant problems. Joint hypermobility can also present as chronic fatigue in children and adults and, again, referral to the physiotherapist is recommended for management.

### Other skeletal manifestations

Many of the skeletal abnormalities seen with VPA exposure are minor and do not require treatment. These include postural talipes, and overlapping or hypoplastic toes [6, 12, 39, 51, 114]. More significant limb defects, principally radial ray defects, tend to occur in those exposed to higher doses of VPA. These can vary in severity, but even mild defects can cause some functional impairment, e.g. with writing. It is important, therefore, to look for minor signs such as flattening of the thenar eminence and check function, with referral to an occupational therapist or hand surgeon if there are significant problems. Talipes which is structural or does not respond to conservative treatment should be managed by a specialist surgeon. Where talipes is resistant to treatment, where there is a sacral dermal sinus or a large or deep dimple, or where there are abnormal neurological signs in the lower limbs, imaging of the spine is recommended [69, 75].

### Adults with fetal valproate Spectrum disorder

Few large studies have been carried out on adolescents and adults with FVSD, and much of the information is limited to anecdotal reports, clinical experience and unpublished data. Bromley et al. have studied intellectual functioning in 18 individuals over the age of 16 with a confirmed diagnosis of FVS [26] and identified increased rates of intellectual disability (IQ < 70), with poor verbal comprehension and reasoning, impaired auditory working memory and processing speed deficits, providing evidence that the neurodevelopmental deficits are persistent into adulthood. Information gathered from individual families support the fact that difficulties continue into adult life, affecting independence and employment opportunities as well as mental health and ability to form relationships. However, it has not been possible to compare incidences with normal population controls. At the present time there does not appear to be an increased incidence of any specific adult medical disorders, though long term effects of congenital malformations and sequelae of joint hypermobility can remain problematic. Weight increase, as noted above, may be an associated feature but has not yet been studied formally. In most cases the family doctor or general practitioner will be the person responsible for care of adults who were exposed to VPA in utero. In some countries there is national guidance for carrying out annual health checks on adults with learning disability [81] and the checklist (Additional file 3) summarises the points which need to be addressed specifically by the GP in older patients with FVSD.

### Transition to adult care

Parents identified the stage of transition to adult care between the ages of 16 and 20 as a particularly problematic time. In many countries plans for special educational needs can continue through school into college and University and thus they can continue to be of benefit. Moving to a suitable college may require travelling further each day and this might pose logistical issues, especially if individuals’ cognitive organisational skills are affected, and it could also incur extra costs. These issues are of great importance to families and should be addressed at the time of transition. Transfer of medical care from the paediatrician to the family doctor occurs in the transitional period and family doctors need to be provided with clear and consistent information as to what health surveillance adults will need as many will be unfamiliar with FVSD.

## Recommendations for the management of FVSD

The recommendations for management of FVSD are given in Table 5. Short versions of these recommendations, tailored to specific groups are included as Additional files 1, 2, 3, 4 and 5.

## Facilitators and barriers to application of the consensus recommendations

This consensus document was drawn up by an expert group covering several different European member states, and reviewing the world-wide literature. The aim is that it will be used in different countries where it is likely that there will be differing access to health care, and different provision of support for development and education. When drawing up the recommendations we had to take this into account and in some areas this meant we needed to be less specific and more general in our recommendations as a result. To overcome this barrier it may be possible in the future to produce versions of the care plan which do not differ in their core recommendations but provide additional information tailored to a specific healthcare system or country, translating the documents into the language required.

The consensus document is a lengthy one and many parents and professionals with busy clinical or other commitments may prefer a short summary. The group felt strongly that shorter summary documents, tailored to different target groups, should be prepared alongside the full consensus paper. This was also favoured by the parent groups. We thus produced one-page summary sheets specifically for parents, the paediatric team, general practitioners who will be overseeing adult care and teachers. These are appended as Additional files 1, 2, 3 and 4. We have put references or links in the documents to existing resources which will be of help to those implementing the consensus recommendations, for example to templates used by general practitioners to undertake an adult learning disability health check [12], and information on how to assess joint hypermobility using the Beighton score [79].

## Resource implications of the recommendations

Though adverse effects of VPA exposure were first described in the literature in the 1980s, recognition of VPA teratogenic effects has taken time [115], especially as there is no diagnostic biomarker for FVSD and, where development is concerned, there are many confounding factors which could be contributing to developmental problems including other drug exposures, seizures during pregnancy, and unknown genetic factors. The spectrum of problems caused by exposure to VPA in pregnancy was not specifically acknowledged as an entity until the results of larger prospective studies started to become available, and this has meant that few individuals with this condition have had any coordinated management in the past. Implementing a new health and developmental surveillance system for this group will therefore, most likely have resource implications. We know, however, from the studies by Adab [98] and Baker [21], that a large proportion of children are receiving additional educational input already, so these costs may not increase unduly. There is no specific drug treatment for the disorder, so we do not anticipate that use of specific drugs will increase dramatically as a result of our recommendations, although methylphenidate may be prescribed with increased frequency if ADHD is recognised more frequently [106]. Where resource implications will have most impact is in the need for monitoring of development and for neuropsychological assessments throughout childhood and early adulthood, interventions which were not previously always carried out. These are offset by the fact that if children with FVSD are provided with the right sort of support they may do better developmentally, behaviourally and socially, and thus pose less of an economic cost to society later on. The very firm guidance on avoidance of VPA treatment during pregnancy published by the EMA in 2018 [2] will most likely lead to a continuing drop in the number of children with FVSD, and we anticipate that it will become a very rare occurrence in the future. Thus, whilst we consider it important for the cohort of existing individuals to have high quality care as outlined in the recommendations, we hope and accept that the need for these will decline as the years go by.

Evaluating how the recommendations within this care plan are being applied will encourage good practice and will also have potential to provide important feedback which will be used to improve patient care. We have thus tried to formulate our recommendations in such a way that they can be used as standards for audit, e.g. phrasing these as “a developmental assessment using a validated tool should be carried out between 6 and 12 months”. We aimed to have criteria that could be used across different countries, accepting that there will need to be some modifications. We aim to undertake a first audit using these criteria 12 months after their publication.

Development of these recommendations drew on the existing work carried out by Dr. Hubert Journel and Professor Sylvie Odent in production of the French PNDS document [30] funded by the French government and on the existing research undertaken in the field by several of the experts concerned. The work on the consensus document was independent of this previous research, though drew on findings from it. The work of the group was facilitated through the European Reference Network ERN-ITHACA which is funded by a CHAFEA grant 769,045. Administrative support and resources for the consensus working group meeting were provided from this grant. No specific funding has been received from industry or other sources. All contributing working group members were asked to declare any possible conflicts of interest.

## Conclusions

These recommendations provide a framework for the diagnosis and management of FVSD. In view of the multisystem involvement it is important that a lead clinician is identified to coordinate care. This is likely to be a community paediatrician during the childhood years but general practitioners/family doctors will have an increasing role to play as this cohort of exposed individuals reach adulthood. These recommendations aim to be pragmatic so that they can be followed in different healthcare systems but it is likely that there will be differences in provision of care between different countries which will need to be taken into consideration. As many of the individuals concerned are currently not having any health surveillance, implementing these recommendations may incur some costs, yet we believe that good quality care will overall improve quality of life, prevent complications and enable affected individuals to achieve their full potential, thus ultimately benefiting themselves and society as a whole. The recommendations will be updated on a two-yearly basis by consensus conference between members of the working group.

## Additional files

Additional file 1: Summary sheet for Patients and Parents. (PPTX 102 kb)

Additional file 2: Summary sheet for Paediatricians and Other Health Professionals. (PPTX 99 kb)

Additional file 3: Summary sheet for General Practitioners. (PPTX 115 kb)

Additional file 4: Summary sheet for Educators. (PPTX 114 kb)

Additional file 5: Summary sheet for Psychologists. (PPTX 102 kb)

## Abbreviations

ADHD: Attention-deficit hyperactivity disorder
AED: Antiepileptic drug
ASD: Autism spectrum disorder
CHAFEA: Consumers, Health, Agriculture and Foods Executive Agency
EMA: European Medicines Agency
e-PAG: European Patient Advocacy Group
ERN: European Reference Network
EUROCAT: European Surveillance of Congenital Anomalies
FVS: Fetal Valproate Syndrome
FVSD: Fetal Valproate Spectrum Disorder
GP: General Practitioner
GU: Genitourinary
MHRA: Medicines and Healthcare Products Regulatory Agency
NEAD: Neurodevelopment after Exposure to Antiepileptic Drugs
PNDS: Protocole Nationale de Diagnostique et de Soin
RCT: Randomised Controlled Trial
SLCN: Speech and Language Communication Needs
SLT: Speech and Language Therapist
VPA: Valproic acid
WWE: Women With Epilepsy

## Authors’ information

This project was only possible through a multidisciplinary approach. JCS, PT and JD are clinical geneticists with a long term interest in teratogenic effects of antiepileptic drugs. RB is a clinical psychologist and AW a developmental neuropsychologist with extensive clinical and research interests in this area. RD is a general practitioner looking after children and adults with learning disability in primary care. RD also has experience in dysmorphology and clinical genetics. VC is a senior physiotherapist who runs a specialist clinic for children with joint hypermobility. HM has a background in teratology and LB is a consultant paediatrician with a special interest in neural tube defects. GJ is a craniofacial surgeon who works in a specialist craniosynostosis team and IB is an academic ENT surgeon. RW is a consultant anaesthetist and AJ is a junior medical doctor who has undertaken previous research studies on the clinical and genetic aspects of Fetal Valproate Syndrome. CD is a speech and language therapist attached to one of the UK Cleft Networks and working in the community. LH and NA are child and adolescent psychiatrists, LH has a specific interest in autism spectrum disorder. JA is a paediatric ophthalmologist and DH is a consultant paediatrician with a special interest in neurodisability. SS is a clinical fellow in paediatric nephrology. GAD is a consultant neonatologist with a specific interest in congenital malformations and dysmorphology. EL is a paediatric cardiologist who has published in this area previously, MR is a research associate who works as data manager for the European Reference Network ERN ITHACA. This project came out of initial work by HJ and SO, clinical geneticists who prepared the French protocol for diagnosis and care on Fetal Valproate Syndrome. Finally, a very important contribution to this group came from JW who is a parent and coordinates a support group for families who have children affected by teratogenic effects of antiepileptic drugs.
