# Supplementary material for: Diagnosis and management of individuals with Fetal Valproate Spectrum Disorder; a consensus statement from the European Reference Network for Congenital Malformations and Intellectual Disability
Source: Orphanet J Rare Dis. 2019 Jul 19;14:180. doi: 10.1186/s13023-019-1064-y (PMC6642533; doi:10.1186/s13023-019-1064-y)
Supplement: Supplementary file 1 — Summary sheet for Patients and Parents. (PPTX 102 kb) [file 13023_2019_1064_MOESM1_ESM.pptx]

## Slide 1
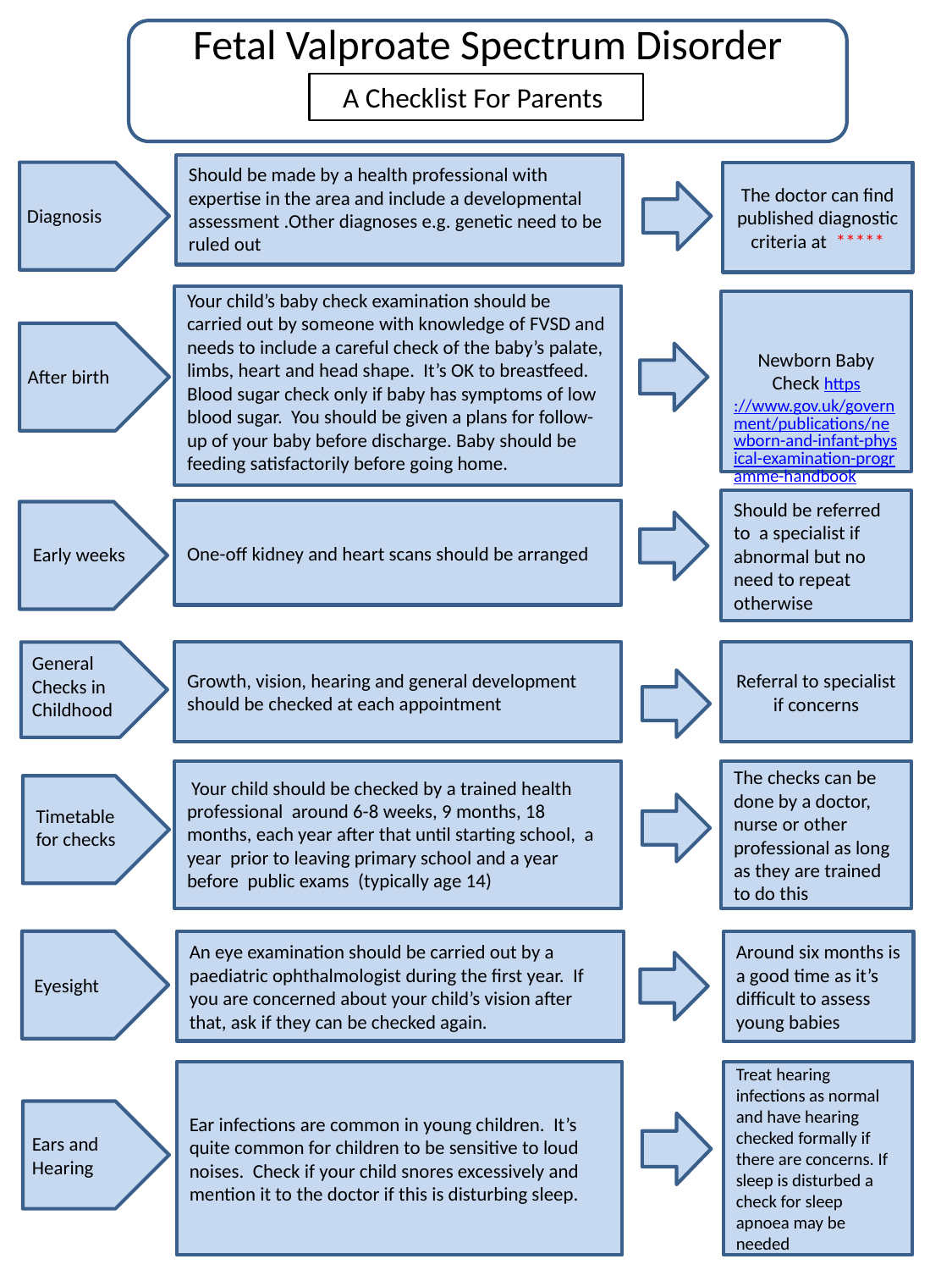

# Fetal Valproate Spectrum Disorder
A Checklist For Parents
Should be made by a health professional with expertise in the area and include a developmental assessment .Other diagnoses e.g. genetic need to be ruled out
The doctor can find published diagnostic criteria at *****
Diagnosis
Your child’s baby check examination should be carried out by someone with knowledge of FVSD and needs to include a careful check of the baby’s palate, limbs, heart and head shape. It’s OK to breastfeed. Blood sugar check only if baby has symptoms of low blood sugar. You should be given a plans for follow-up of your baby before discharge. Baby should be feeding satisfactorily before going home.
Newborn Baby Check https://www.gov.uk/government/publications/newborn-and-infant-physical-examination-programme-handbook
After birth
Should be referred to a specialist if abnormal but no need to repeat otherwise
One-off kidney and heart scans should be arranged
Early weeks
Growth, vision, hearing and general development should be checked at each appointment
Referral to specialist if concerns
General Checks in Childhood
 Your child should be checked by a trained health professional around 6-8 weeks, 9 months, 18 months, each year after that until starting school, a year prior to leaving primary school and a year before public exams (typically age 14)
The checks can be done by a doctor, nurse or other professional as long as they are trained to do this
Timetable
for checks
An eye examination should be carried out by a paediatric ophthalmologist during the first year. If you are concerned about your child’s vision after that, ask if they can be checked again.
Around six months is a good time as it’s difficult to assess young babies
Eyesight
Ear infections are common in young children. It’s quite common for children to be sensitive to loud noises. Check if your child snores excessively and mention it to the doctor if this is disturbing sleep.
Treat hearing infections as normal and have hearing checked formally if there are concerns. If sleep is disturbed a check for sleep apnoea may be needed
Ears and
Hearing

## Slide 2
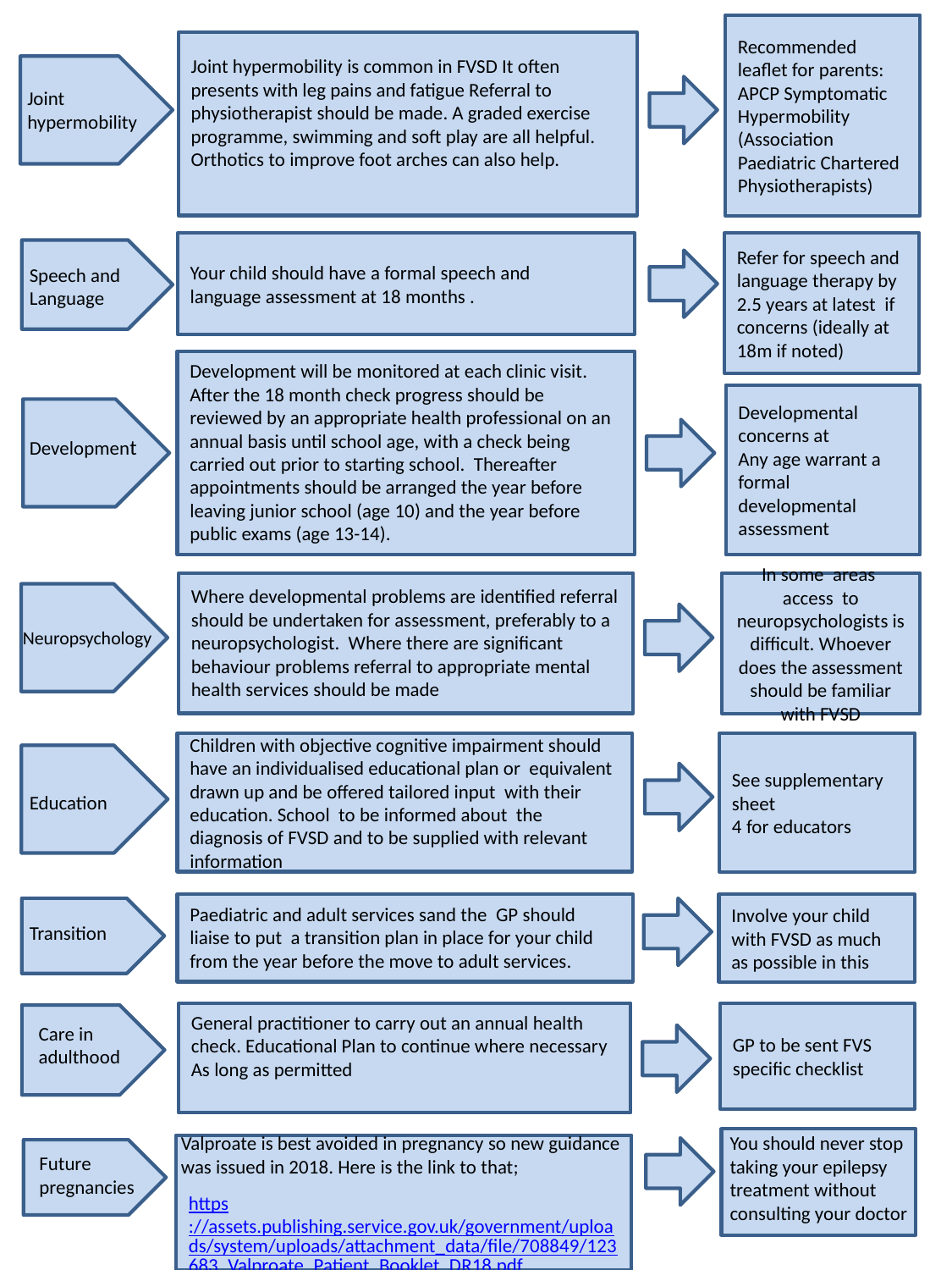

Recommended leaflet for parents:
APCP Symptomatic Hypermobility
(Association Paediatric Chartered
Physiotherapists)
Joint hypermobility is common in FVSD It often presents with leg pains and fatigue Referral to physiotherapist should be made. A graded exercise programme, swimming and soft play are all helpful. Orthotics to improve foot arches can also help.
Joint
hypermobility
Refer for speech and language therapy by 2.5 years at latest if concerns (ideally at 18m if noted)
Your child should have a formal speech and
language assessment at 18 months .
Speech and Language
Development will be monitored at each clinic visit.
After the 18 month check progress should be
reviewed by an appropriate health professional on an annual basis until school age, with a check being carried out prior to starting school. Thereafter appointments should be arranged the year before leaving junior school (age 10) and the year before public exams (age 13-14).
Developmental concerns at
Any age warrant a formal
developmental assessment
Development
In some areas access to neuropsychologists is difficult. Whoever does the assessment should be familiar with FVSD
Where developmental problems are identified referral should be undertaken for assessment, preferably to a neuropsychologist. Where there are significant behaviour problems referral to appropriate mental health services should be made
Neuropsychology
See supplementary sheet
4 for educators
Children with objective cognitive impairment should have an individualised educational plan or equivalent drawn up and be offered tailored input with their education. School to be informed about the diagnosis of FVSD and to be supplied with relevant information
Education
Involve your child with FVSD as much as possible in this
Paediatric and adult services sand the GP should liaise to put a transition plan in place for your child from the year before the move to adult services.
Transition
GP to be sent FVS
specific checklist
General practitioner to carry out an annual health check. Educational Plan to continue where necessary
As long as permitted
Care in adulthood
Valproate is best avoided in pregnancy so new guidance
was issued in 2018. Here is the link to that;
You should never stop
taking your epilepsy
treatment without
consulting your doctor
https://assets.publishing.service.gov.uk/government/uploads/system/uploads/attachment_data/file/708849/123683_Valproate_Patient_Booklet_DR18.pdf
Future
pregnancies
