# Supplementary material for: Diagnosis and management of individuals with Fetal Valproate Spectrum Disorder; a consensus statement from the European Reference Network for Congenital Malformations and Intellectual Disability
Source: Orphanet J Rare Dis. 2019 Jul 19;14:180. doi: 10.1186/s13023-019-1064-y (PMC6642533; doi:10.1186/s13023-019-1064-y)
Supplement: Supplementary file 2 — Summary sheet for Paediatricians and Other Health Professionals. (PPTX 99 kb) [file 13023_2019_1064_MOESM2_ESM.pptx]

## Slide 1
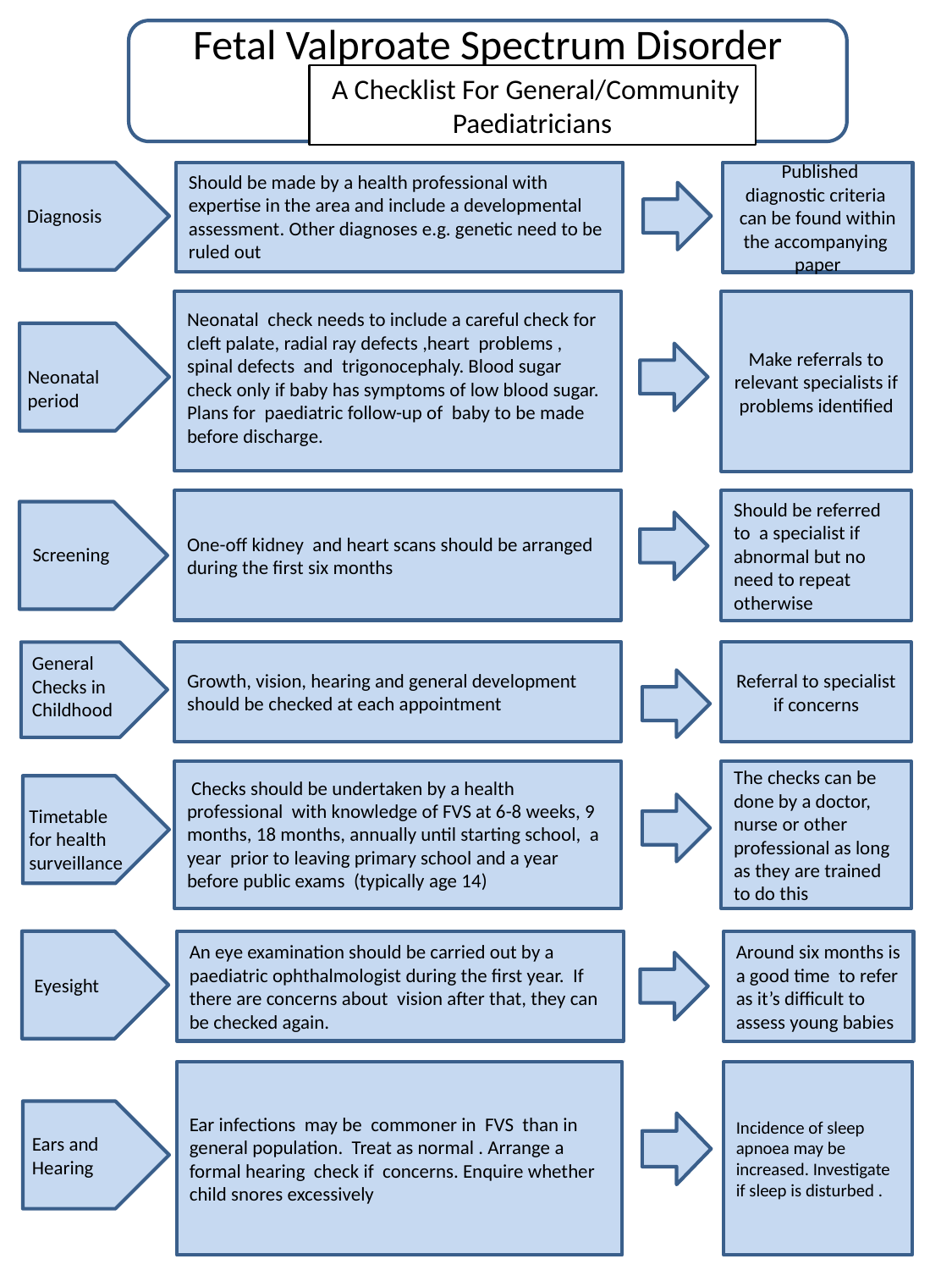

# Fetal Valproate Spectrum Disorder
 A Checklist For General/Community Paediatricians
Should be made by a health professional with expertise in the area and include a developmental assessment. Other diagnoses e.g. genetic need to be ruled out
 Published diagnostic criteria can be found within the accompanying paper
Diagnosis
 Make referrals to relevant specialists if problems identified
Neonatal check needs to include a careful check for cleft palate, radial ray defects ,heart problems , spinal defects and trigonocephaly. Blood sugar check only if baby has symptoms of low blood sugar. Plans for paediatric follow-up of baby to be made before discharge.
Neonatal period
One-off kidney and heart scans should be arranged during the first six months
Should be referred to a specialist if abnormal but no need to repeat otherwise
Screening
Growth, vision, hearing and general development should be checked at each appointment
Referral to specialist if concerns
General Checks in Childhood
 Checks should be undertaken by a health professional with knowledge of FVS at 6-8 weeks, 9 months, 18 months, annually until starting school, a year prior to leaving primary school and a year before public exams (typically age 14)
The checks can be done by a doctor, nurse or other professional as long as they are trained to do this
Timetable
for health
surveillance
An eye examination should be carried out by a paediatric ophthalmologist during the first year. If there are concerns about vision after that, they can be checked again.
Around six months is a good time to refer as it’s difficult to assess young babies
Eyesight
Ear infections may be commoner in FVS than in general population. Treat as normal . Arrange a formal hearing check if concerns. Enquire whether child snores excessively
Incidence of sleep apnoea may be increased. Investigate if sleep is disturbed .
Ears and
Hearing

## Slide 2
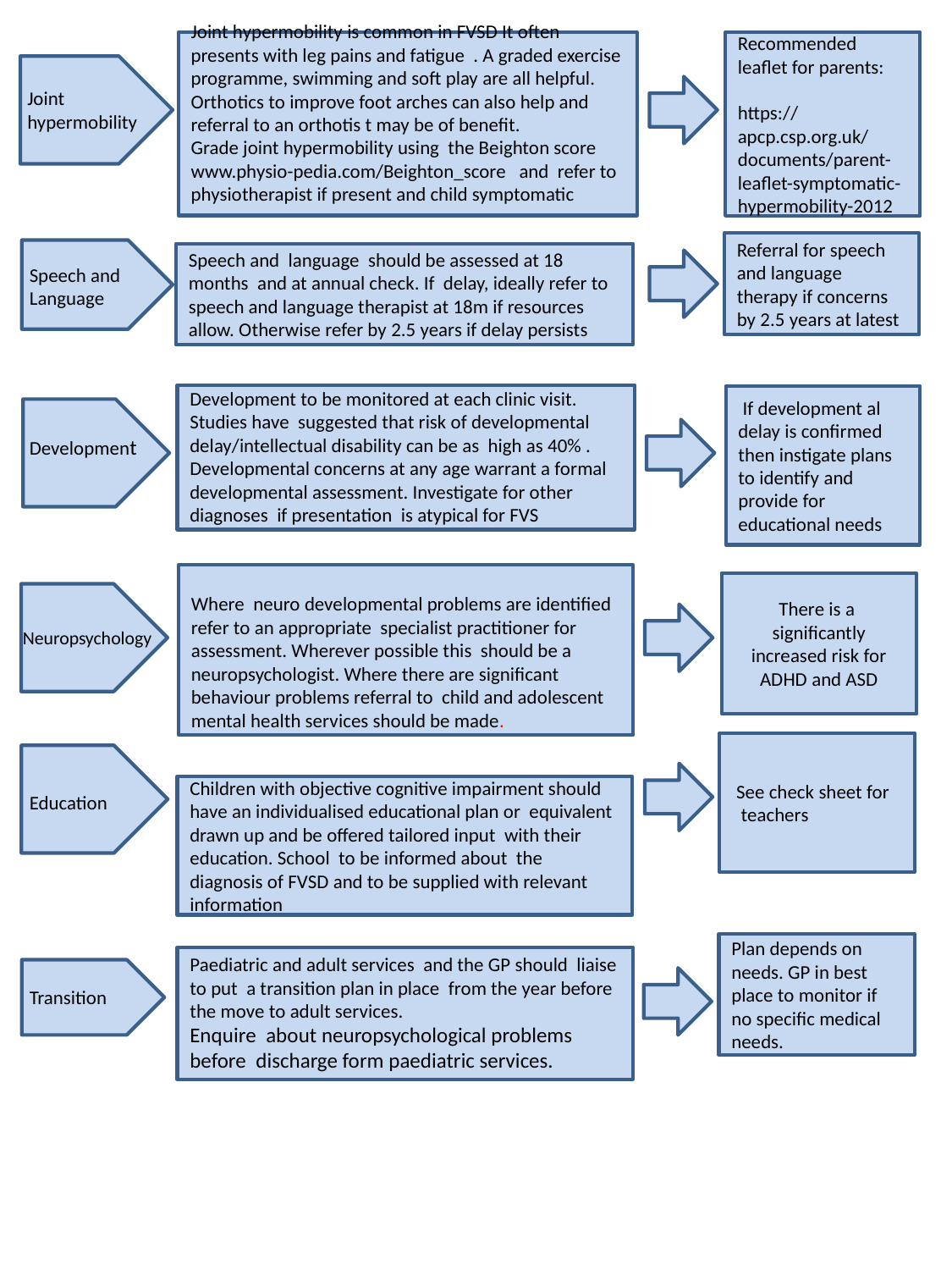

Joint hypermobility is common in FVSD It often presents with leg pains and fatigue . A graded exercise programme, swimming and soft play are all helpful. Orthotics to improve foot arches can also help and referral to an orthotis t may be of benefit.
Grade joint hypermobility using the Beighton score www.physio-pedia.com/Beighton_score and refer to physiotherapist if present and child symptomatic
Recommended leaflet for parents:
https://apcp.csp.org.uk/documents/parent-leaflet-symptomatic-hypermobility-2012
Joint
hypermobility
Referral for speech and language
therapy if concerns by 2.5 years at latest
Speech and language should be assessed at 18 months and at annual check. If delay, ideally refer to speech and language therapist at 18m if resources allow. Otherwise refer by 2.5 years if delay persists
Speech and Language
Development to be monitored at each clinic visit.
Studies have suggested that risk of developmental delay/intellectual disability can be as high as 40% . Developmental concerns at any age warrant a formal developmental assessment. Investigate for other diagnoses if presentation is atypical for FVS
 If development al delay is confirmed then instigate plans to identify and provide for educational needs
Development
Where neuro developmental problems are identified refer to an appropriate specialist practitioner for assessment. Wherever possible this should be a neuropsychologist. Where there are significant behaviour problems referral to child and adolescent mental health services should be made.
There is a significantly increased risk for ADHD and ASD
Neuropsychology
 See check sheet for
 teachers
Children with objective cognitive impairment should have an individualised educational plan or equivalent drawn up and be offered tailored input with their education. School to be informed about the diagnosis of FVSD and to be supplied with relevant information
Education
Plan depends on needs. GP in best place to monitor if no specific medical needs.
Paediatric and adult services and the GP should liaise to put a transition plan in place from the year before the move to adult services.
Enquire about neuropsychological problems before discharge form paediatric services.
Transition
