# Supplementary material for: Diagnosis and management of individuals with Fetal Valproate Spectrum Disorder; a consensus statement from the European Reference Network for Congenital Malformations and Intellectual Disability
Source: Orphanet J Rare Dis. 2019 Jul 19;14:180. doi: 10.1186/s13023-019-1064-y (PMC6642533; doi:10.1186/s13023-019-1064-y)
Supplement: Supplementary file 3 — Summary sheet for General Practitioners. (PPTX 115 kb) [file 13023_2019_1064_MOESM3_ESM.pptx]

## Slide 1
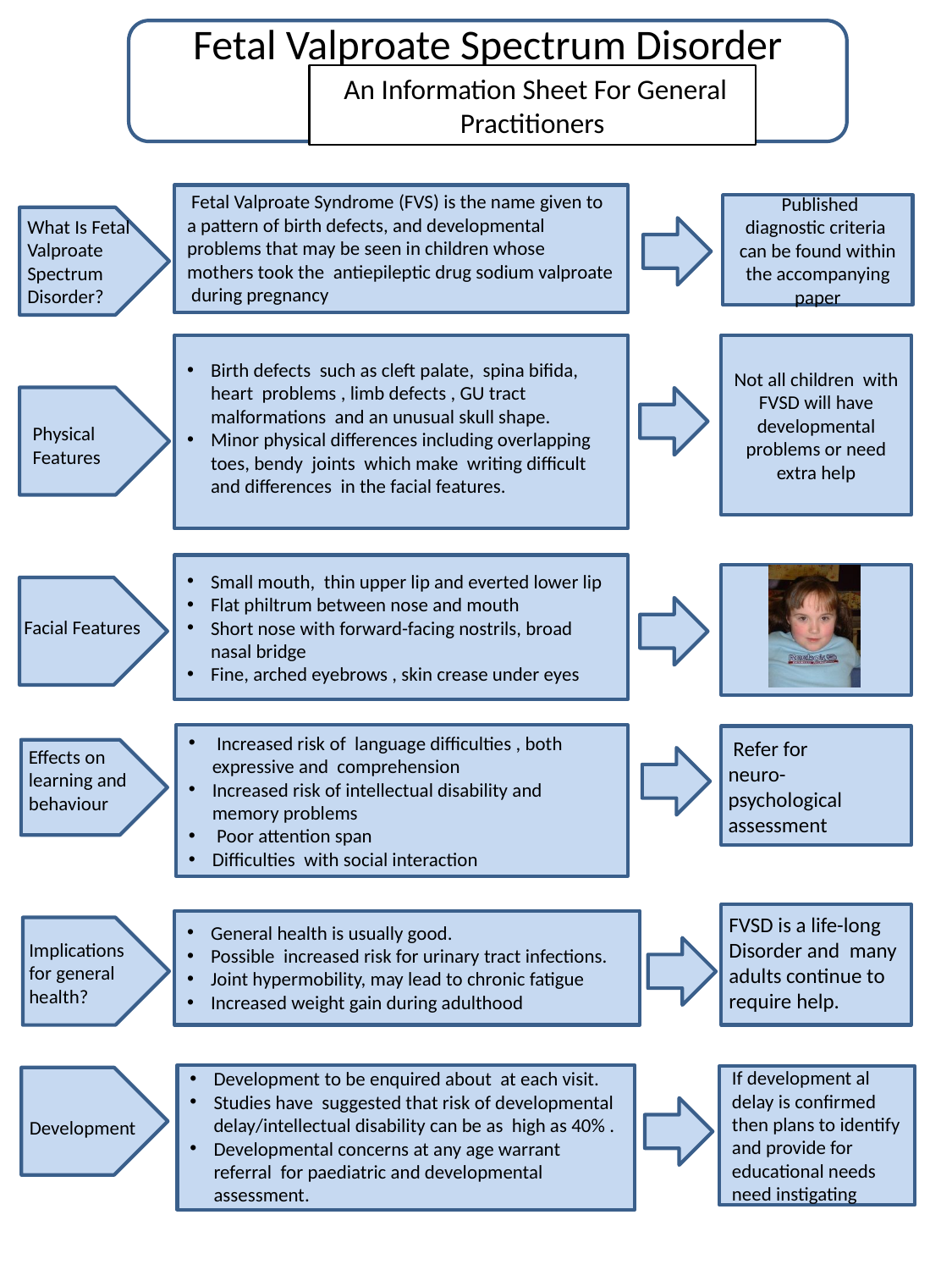

# Fetal Valproate Spectrum Disorder
 An Information Sheet For General Practitioners
 Fetal Valproate Syndrome (FVS) is the name given to a pattern of birth defects, and developmental problems that may be seen in children whose mothers took the antiepileptic drug sodium valproate during pregnancy
 Published diagnostic criteria can be found within the accompanying paper
What Is Fetal
Valproate
Spectrum
Disorder?
Birth defects such as cleft palate, spina bifida, heart problems , limb defects , GU tract malformations and an unusual skull shape.
Minor physical differences including overlapping toes, bendy joints which make writing difficult and differences in the facial features.
Not all children with FVSD will have developmental problems or need extra help
Physical Features
Small mouth, thin upper lip and everted lower lip
Flat philtrum between nose and mouth
Short nose with forward-facing nostrils, broad nasal bridge
Fine, arched eyebrows , skin crease under eyes
 Facial Features
 Increased risk of language difficulties , both expressive and comprehension
Increased risk of intellectual disability and memory problems
 Poor attention span
Difficulties with social interaction
 Refer for
neuro-
psychological
assessment
Effects on
learning and
behaviour
FVSD is a life-long
Disorder and many
adults continue to
require help.
Implications
for general
health?
General health is usually good.
Possible increased risk for urinary tract infections.
Joint hypermobility, may lead to chronic fatigue
Increased weight gain during adulthood
Development to be enquired about at each visit.
Studies have suggested that risk of developmental delay/intellectual disability can be as high as 40% .
Developmental concerns at any age warrant referral for paediatric and developmental assessment.
If development al delay is confirmed then plans to identify and provide for educational needs need instigating
Development

## Slide 2
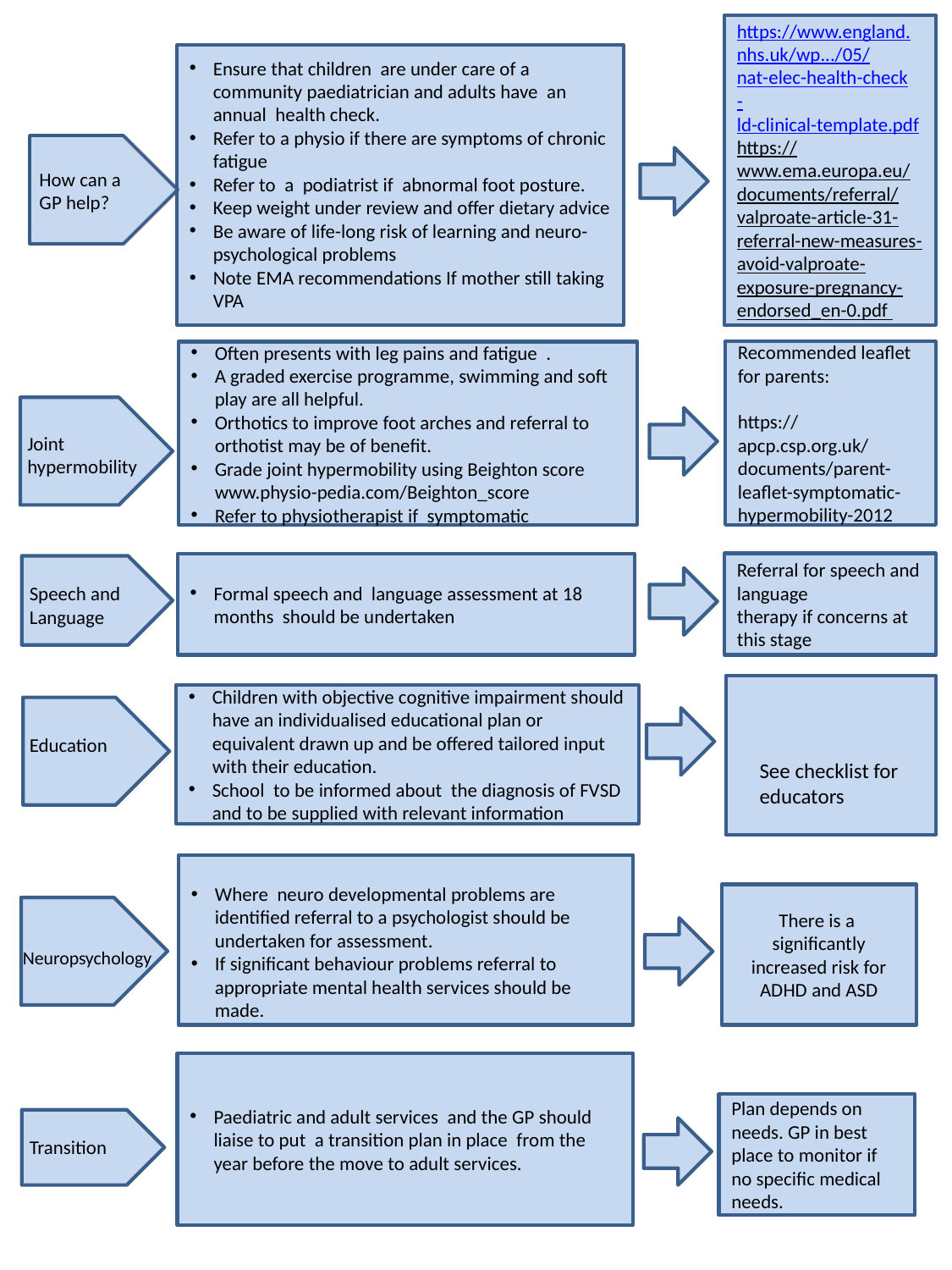

https://www.england.
nhs.uk/wp.../05/
nat-elec-health-check
-ld-clinical-template.pdf
https://www.ema.europa.eu/documents/referral/valproate-article-31-referral-new-measures-avoid-valproate-exposure-pregnancy-endorsed_en-0.pdf
Ensure that children are under care of a community paediatrician and adults have an annual health check.
Refer to a physio if there are symptoms of chronic fatigue
Refer to a podiatrist if abnormal foot posture.
Keep weight under review and offer dietary advice
Be aware of life-long risk of learning and neuro-psychological problems
Note EMA recommendations If mother still taking VPA
How can a
GP help?
Recommended leaflet for parents:
https://apcp.csp.org.uk/documents/parent-leaflet-symptomatic-hypermobility-2012
Often presents with leg pains and fatigue .
A graded exercise programme, swimming and soft play are all helpful.
Orthotics to improve foot arches and referral to orthotist may be of benefit.
Grade joint hypermobility using Beighton score www.physio-pedia.com/Beighton_score
Refer to physiotherapist if symptomatic
Joint
hypermobility
Referral for speech and language
therapy if concerns at this stage
Formal speech and language assessment at 18 months should be undertaken
Speech and Language
Children with objective cognitive impairment should have an individualised educational plan or equivalent drawn up and be offered tailored input with their education.
School to be informed about the diagnosis of FVSD and to be supplied with relevant information
Education
See checklist for
educators
Where neuro developmental problems are identified referral to a psychologist should be undertaken for assessment.
If significant behaviour problems referral to appropriate mental health services should be made.
There is a significantly increased risk for ADHD and ASD
Neuropsychology
Paediatric and adult services and the GP should liaise to put a transition plan in place from the year before the move to adult services.
Plan depends on needs. GP in best place to monitor if no specific medical needs.
Transition
