# Supplementary material for: Diagnosis and management of individuals with Fetal Valproate Spectrum Disorder; a consensus statement from the European Reference Network for Congenital Malformations and Intellectual Disability
Source: Orphanet J Rare Dis. 2019 Jul 19;14:180. doi: 10.1186/s13023-019-1064-y (PMC6642533; doi:10.1186/s13023-019-1064-y)
Supplement: Supplementary file 4 — Summary sheet for Educators. (PPTX 114 kb) [file 13023_2019_1064_MOESM4_ESM.pptx]

## Slide 1
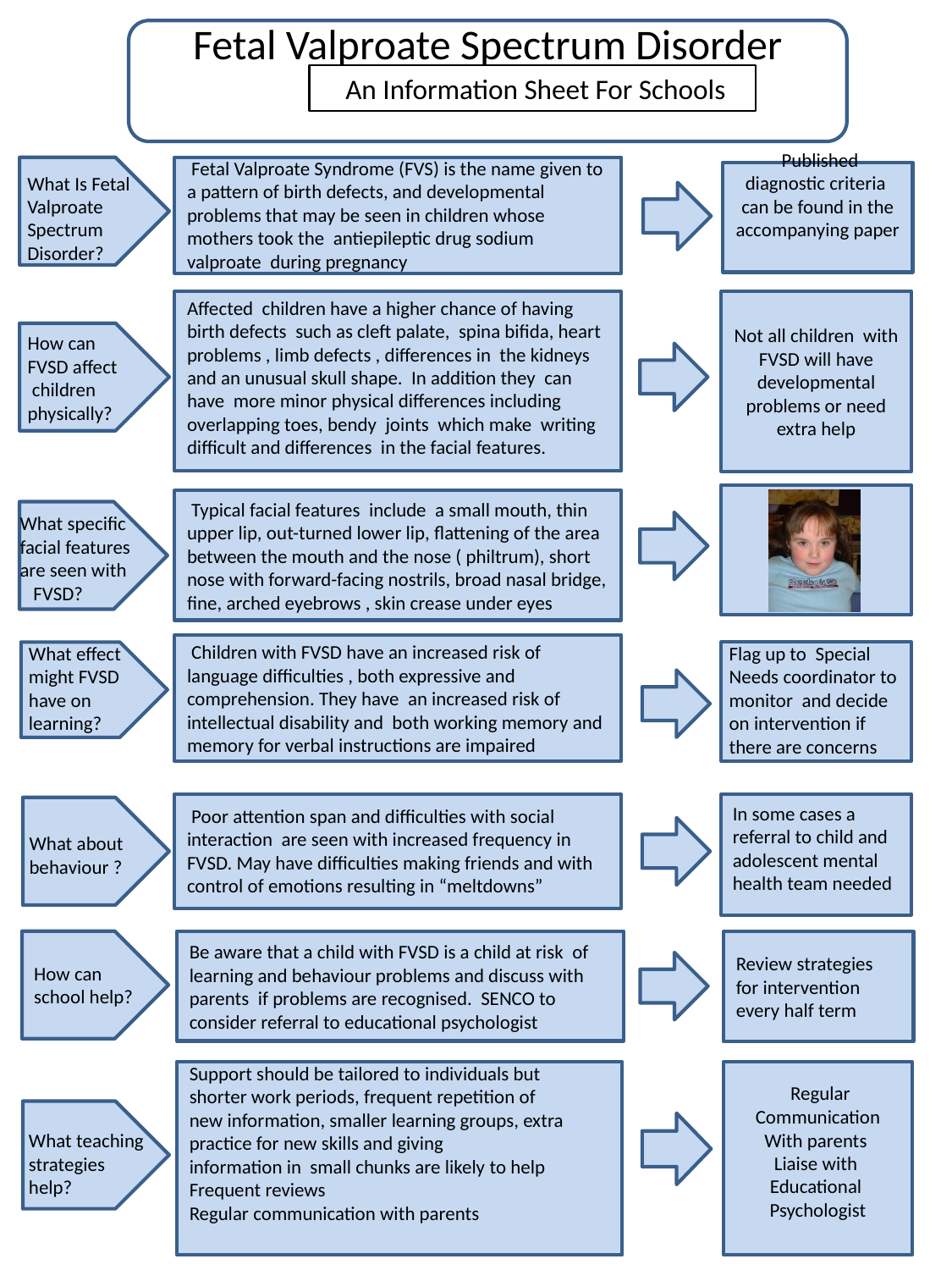

# Fetal Valproate Spectrum Disorder
 An Information Sheet For Schools
 Fetal Valproate Syndrome (FVS) is the name given to a pattern of birth defects, and developmental problems that may be seen in children whose mothers took the antiepileptic drug sodium valproate during pregnancy
 Published diagnostic criteria can be found in the accompanying paper
What Is Fetal
Valproate
Spectrum
Disorder?
Not all children with FVSD will have developmental problems or need extra help
Affected children have a higher chance of having birth defects such as cleft palate, spina bifida, heart problems , limb defects , differences in the kidneys and an unusual skull shape. In addition they can have more minor physical differences including overlapping toes, bendy joints which make writing difficult and differences in the facial features.
How can FVSD affect children physically?
 Typical facial features include a small mouth, thin upper lip, out-turned lower lip, flattening of the area between the mouth and the nose ( philtrum), short nose with forward-facing nostrils, broad nasal bridge, fine, arched eyebrows , skin crease under eyes
What specific
facial features
are seen with
 FVSD?
What effect
might FVSD have on
learning?
 Children with FVSD have an increased risk of language difficulties , both expressive and comprehension. They have an increased risk of intellectual disability and both working memory and memory for verbal instructions are impaired
Flag up to Special
Needs coordinator to
monitor and decide
on intervention if
there are concerns
 Poor attention span and difficulties with social interaction are seen with increased frequency in FVSD. May have difficulties making friends and with control of emotions resulting in “meltdowns”
In some cases a
referral to child and
adolescent mental
health team needed
What about
behaviour ?
Be aware that a child with FVSD is a child at risk of learning and behaviour problems and discuss with parents if problems are recognised. SENCO to consider referral to educational psychologist
Review strategies for intervention every half term
 How can
 school help?
Support should be tailored to individuals but
shorter work periods, frequent repetition of
new information, smaller learning groups, extra
practice for new skills and giving
information in small chunks are likely to help
Frequent reviews
Regular communication with parents
 Regular
Communication
With parents
Liaise with
Educational
Psychologist
What teaching
strategies
help?
