# Supplementary material for: Diagnosis and management of individuals with Fetal Valproate Spectrum Disorder; a consensus statement from the European Reference Network for Congenital Malformations and Intellectual Disability
Source: Orphanet J Rare Dis. 2019 Jul 19;14:180. doi: 10.1186/s13023-019-1064-y (PMC6642533; doi:10.1186/s13023-019-1064-y)
Supplement: Supplementary file 5 — Summary sheet for Psychologists. (PPTX 102 kb) [file 13023_2019_1064_MOESM5_ESM.pptx]

## Slide 1
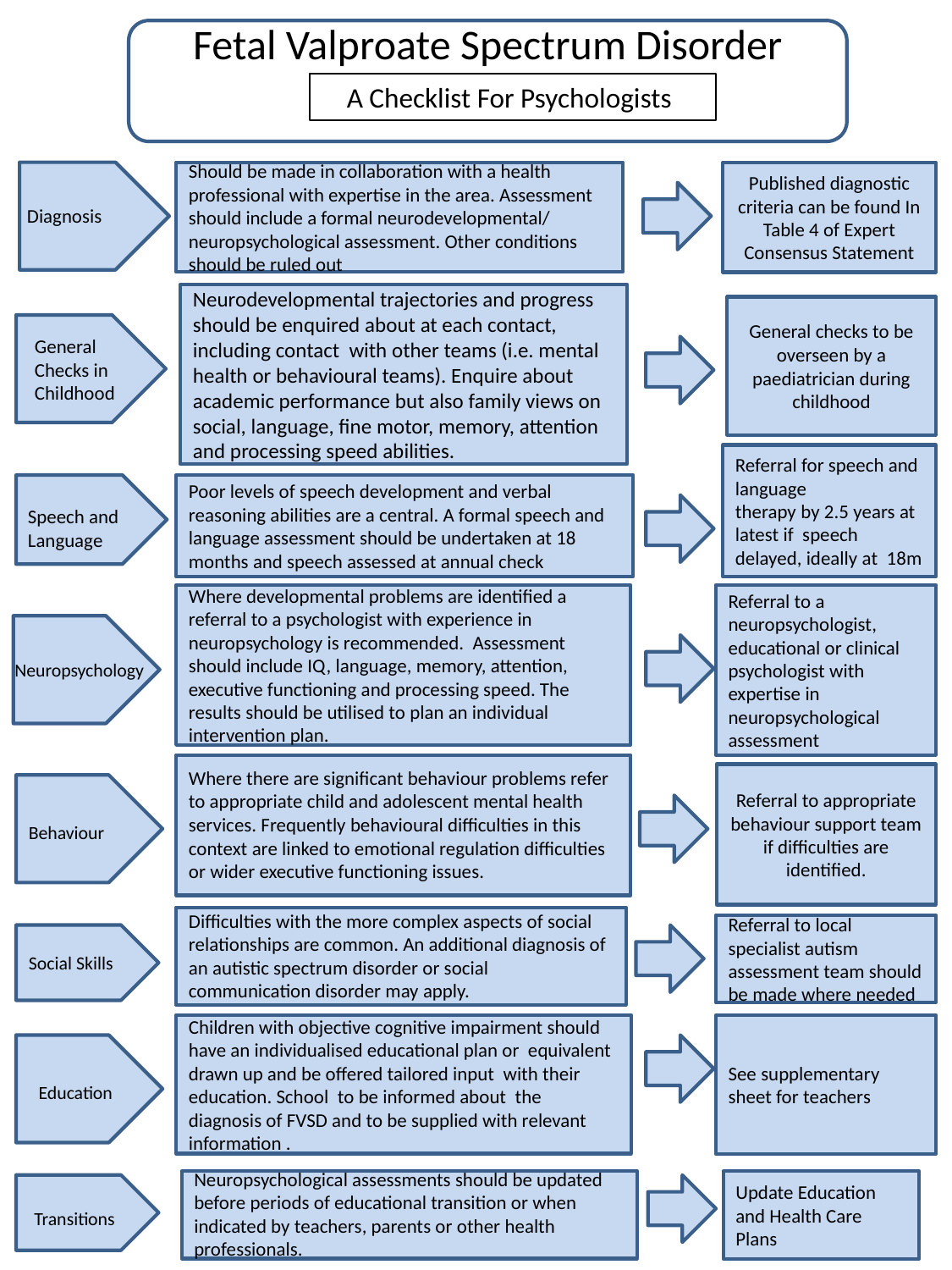

# Fetal Valproate Spectrum Disorder
A Checklist For Psychologists
Should be made in collaboration with a health professional with expertise in the area. Assessment should include a formal neurodevelopmental/ neuropsychological assessment. Other conditions should be ruled out
Published diagnostic criteria can be found In Table 4 of Expert Consensus Statement
Diagnosis
Neurodevelopmental trajectories and progress should be enquired about at each contact, including contact with other teams (i.e. mental health or behavioural teams). Enquire about academic performance but also family views on social, language, fine motor, memory, attention and processing speed abilities.
General checks to be overseen by a paediatrician during childhood
General Checks in Childhood
Referral for speech and language
therapy by 2.5 years at latest if speech delayed, ideally at 18m
Poor levels of speech development and verbal reasoning abilities are a central. A formal speech and
language assessment should be undertaken at 18 months and speech assessed at annual check
Speech and Language
Where developmental problems are identified a referral to a psychologist with experience in neuropsychology is recommended. Assessment should include IQ, language, memory, attention, executive functioning and processing speed. The results should be utilised to plan an individual intervention plan.
Referral to a neuropsychologist, educational or clinical psychologist with expertise in neuropsychological assessment
Neuropsychology
Where there are significant behaviour problems refer to appropriate child and adolescent mental health services. Frequently behavioural difficulties in this context are linked to emotional regulation difficulties or wider executive functioning issues.
Referral to appropriate behaviour support team if difficulties are identified.
Behaviour
Difficulties with the more complex aspects of social relationships are common. An additional diagnosis of an autistic spectrum disorder or social communication disorder may apply.
Referral to local specialist autism assessment team should be made where needed
Social Skills
Children with objective cognitive impairment should have an individualised educational plan or equivalent drawn up and be offered tailored input with their education. School to be informed about the diagnosis of FVSD and to be supplied with relevant information .
See supplementary sheet for teachers
Education
Update Education and Health Care Plans
Neuropsychological assessments should be updated before periods of educational transition or when indicated by teachers, parents or other health professionals.
Transitions
